# Boosting Mechanoluminescence Performance in Doped CaZnOS by the Facile Self‐Reduction Approach

**DOI:** 10.1002/adma.202511643

**Published:** 2025-09-26

**Authors:** Shengbin Xu, Yao Xiao, Puxian Xiong, Pan Zheng, Sheng Wu, Xuesong Wang, Yumin Yin, Haiqiang Fang, Chengan Wang, Yuexi Lu, Enhai Song, Jiulin Gan

**Affiliations:** ^1^ State Key Laboratory of Luminescent Materials and Devices Institute of Optical Communication Materials Guangdong Engineering Technology Research and Development Center of Special Optical Fiber Materials and Devices Guangdong Provincial Key Laboratory of Fiber Laser Materials and Applied Techniques South China University of Technology Guangzhou 510640 China; ^2^ Department of Electrical and Electronic Engineering The University of Hong Kong Hong Kong 999077 China; ^3^ Guangdong Basic Research Center of Excellence for Structure and Fundamental Interactions of Matter Guangdong Provincial Key Laboratory of Quantum Engineering and Quantum Materials Guangdong‐Hong Kong Joint Laboratory of Quantum Matter Frontier Research Institute for Physics School of Physics South China Normal University Guangzhou 510006 China

**Keywords:** CaZnOS, lattice defect, mechanoluminescence, orthodontic sensor, self‐duction

## Abstract

Mechanoluminescence (ML), the emission of light under mechanical stimuli, shows great potential in passive sensing, wearable devices, and biomedical diagnostics. However, the practical application of ML materials is hindered by low intensity and poor self‐recoverable performance. Herein, a Mn^4+^→Mn^2+^ self‐reduction strategy is presented to significantly enhance the self‐recoverable ML performance of CaZnOS by inducing lattice defects and promoting distortion in its noncentrosymmetric hexagonal structure. This approach enhances the internal piezoelectric response and increases the maximum ML intensity up to 4 times. X‐ray absorption near‐edge structure, extended X‐ray absorption fine structure, electron paramagnetic resonance, piezoresponse force microscopy, and density functional theory calculations reveal that the composite defects involving $V_{O}^{\cdot \cdot }$ and 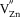 are the key to the significant enhancement of ML. Furthermore, this strategy is successfully extended to rare‐earth ions codoped systems, achieving a general enhancement of near‐infrared ML emission. Based on these findings, a multilayer orthodontic sensor is developed, capable of real‐time occlusal mapping and bite‐force monitoring. The device exhibits sensitive response across 0–12 N and achieves 96.89% accuracy in occlusal localization through neuromorphic image recognition. This work offers a generalizable route toward ML performance optimization and paves the way for the development of advanced intelligent sensing technologies.

## Graphical abstract

Self‐reduction primarily enhances mechanoluminescence intensity by deliberately introducing lattice defects, which increase structural distortion and amplify the piezoelectric response, thereby improving the force‐to‐light conversion efficiency. Additionally, self‐reduction optimizes the local distribution of Mn^2+^ ions, promoting energy concentration within the emission band.

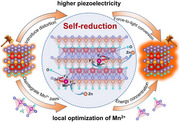

## 1 Introduction

Mechanoluminescence (ML), defined as the direct conversion of mechanical energy into light emission, has emerged as a promising intelligent optical phenomenon with wide‐ranging applications in passive sensing, biomedical imaging, and advanced anticounterfeiting technologies.^[^ ^1^ ^]^ Unlike conventional luminescent materials that require external excitation sources, ML materials can emit photons solely through mechanical stimuli, such as compression or friction.^[^ ^2^ ^]^ This unique property makes them particularly attractive for use in flexible electronics and wearable devices.^[^ ^3^ ^]^ Although various ML systems spanning ultraviolet to near‐infrared  (NIR) spectral ranges have been developed, practical applications remain constrained by key limitations, including low intensity and poor self‐recoverability.^[^ ^4^ ^]^ To address these challenges, recent research has shifted focus toward the development of high‐performance, self‐recoverable ML materials, such as LaAlO_5_:Cr^3+^ and BaSi_2_O_2_N_2_:Eu^2+^.^[^ ^5^ ^]^ However, these materials still have considerable gap when compared to classical ML systems like ZnS and CaZnOS.^[^ ^6^ ^]^ Consequently, instead of designing entirely new ML materials from scratch, improving existing high‐performance ML materials has become a more viable approach.^[^ ^7^ ^]^ In recent years, significant enhancements in ML performance have been achieved, such as through heterojunction engineering, host‐ion substitution and codoping strategies.^[^ ^8^ ^]^ For example, Peng et al. constructed a ZnS/CaZnOS heterojunction, where band‐edge offset facilitated efficient interfacial charge transfer, and this results in ML intensity up to twice that of commercial ZnS.^[^ ^9^ ^]^ Zou et al. enhanced ML output by partially substituting Ca^2+^ with Ba^2+^ and introducing Bi^3+^ dopants in CaZnOS, increasing the intensity by an order of magnitude.^[^ ^10^ ^]^ Zhang et al. further demonstrated that codoping Nd^3+^ by Mn^2+^ enabled energy transfer from Mn^2+^ to Nd^3+^, boosting NIR ML emission by a factor of 2.6.^[^ ^11^ ^]^ Despite these advances, most strategies lack in a solid theoretical foundation and often rely on empirical trial‐and‐error approaches. It remains difficult to accurately predict the structure–property relationships between defect formation and ML performance, with such strategies often being limited to specific dopant–host combinations. Therefore, developing a broadly applicable and reliably predictive strategy for optimizing ML performance remains a key scientific challenge.^[^ ^12^ ^]^

Regarding ML mechanisms, no unified theory currently accounts for all observed phenomena.^[^ ^13^ ^]^ However, for inorganic ML systems, three primary mechanisms are widely recognized: i) defect‐controlled ML, which relies on carrier trapping and release;^[^ ^14^ ^]^ ii) piezoelectric‐induced ML, where carrier detrapping and recombination are driven by piezoelectric polarization;^[^ ^15^ ^]^ and iii) triboelectric‐induced ML, which is based on interfacial charge transfer.^[^ ^16^ ^]^ What is particularly worth noting is that most of the elastic ML materials with self‐recoverable properties are derived from the piezoelectric‐induced excitation mechanism, involving a multistep process: mechanical deformation induces polarization, leading to carrier detrapping, recombination, nonradiative energy transfer, and eventual light emission. This mechanism has been systematically validated, such as in ZnS:Mn^2+^, CaZnOS: Mn^2+^, and LiGa_5_O_8_:Cr^3+^.^[^ ^7^, ^17^ ^]^ Chandra et al. once proposed in 2013 that self‐recoverable ML in sulfide‐based systems is attributed to carrier trapping in piezoelectric fields.^[^ ^18^ ^]^ Wang et al. further described the phenomenon as a bidirectional coupling between mechanical polarization and photon excitation.^[^ ^19^ ^]^ Therefore, incorporating luminescent ions into noncentrosymmetric piezoelectric matrices has always been an important strategy in the development of ML materials.^[^ ^20^ ^]^ In recent years, studies have shown that even originally centrosymmetric matrices can trigger localized nonsymmetrical structures due to the doping of luminescent ions, thereby achieving piezoelectric responses and exhibiting ML behavior.^[^ ^21^ ^]^ Therefore, it is generally believed that the self‐recoverable elastic ML performance is closely related to the intensity of the piezoelectric response of the material, and stronger piezoelectricity usually means stronger ML intensity for the identical material.^[^ ^22^ ^]^

**Figure 1:**
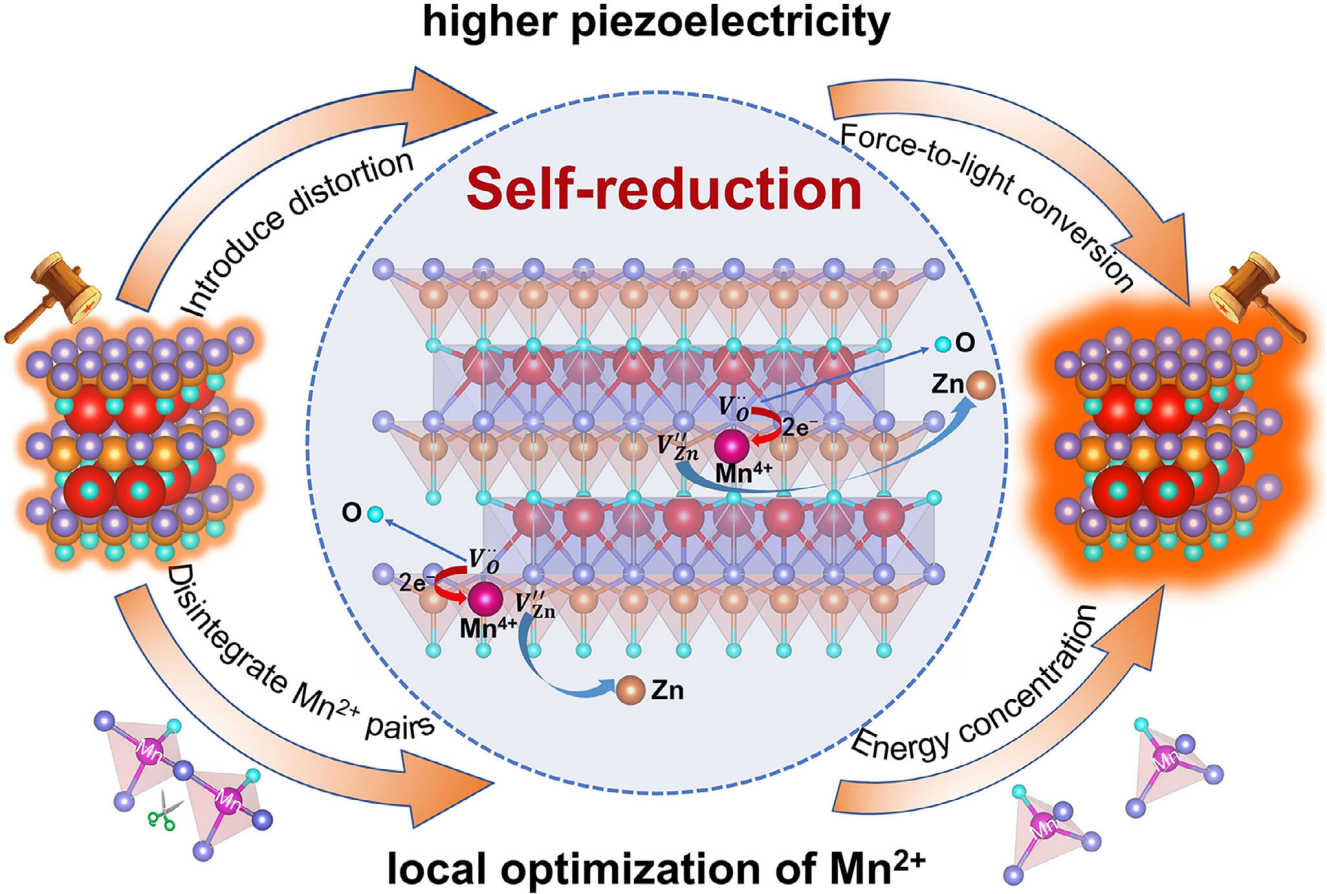
Schematic illustration of ML enhancement via self‐reduction strategy. Self‐reduction primarily enhances ML intensity by deliberately introducing lattice defects, which increase structural distortion and amplify the piezoelectric response, thereby improving the force‐to‐light conversion efficiency. Additionally, self‐reduction optimizes the local distribution of Mn^2+^ ions, promoting energy concentration within the emission band.

Based on the above understandings, we artificially introduce lattice defects in the prototypical piezoelectric ML material CaZnOS through a self‐reduction strategy, inducing significant distortion in its noncentrosymmetric hexagonal phase. This structural perturbation enhances the internal piezoelectric response and remarkably boosts the ML intensity. Unexpectedly, the self‐reduction strategy also optimizes the local distribution of Mn^2+^, resulting in energy concentration on the isolated Mn^2+^, which further enhances the ML intensity. The schematic diagram illustrating the mechanism of the self‐reduction regulation strategy is shown in **Figure** **1**. Subsequently, we further demonstrated that the self‐reduction process can significantly enhance the ML performance in the CaZnOS system doped with rare‐earth (RE) ions. A comprehensive analysis of previously reported self‐reduction systems indicates that materials with 2D layered structures which are analogous to graphene exhibit superior electron transport capabilities (Table S1, Supporting Information).^[^ ^23^ ^]^ CaZnOS, as a widely studied high‐performance ML host, has a similar layered crystal structure, contributing to its efficient self‐reduction behavior. The synergistic interaction between intrinsic defect and an integrated electron transport network enables the spontaneous reduction of high‐valence ions to lower valence states. By simply replacing MnCO_3_ with MnO_2_, we achieved a substantial enhancement of the ML intensity of CaZnOS: Mn^2+^ by a factor of 2.0–5.7, with the maximum reaching 4 times that of the initial intensity. X‐ray absorption near‐edge structure (XANES) analysis confirms the successful reduction of Mn^4+^ to Mn^2+^, while extended X‐ray absorption fine structure (EXAFS) and electron paramagnetic resonance (EPR) spectra reveal the evolution of the Mn^2+^ coordination environment. Piezoresponse force microscopy (PFM) and density functional theory (DFT) calculations provide a systematic framework linking self‐reduction behavior, local lattice distortion, enhanced piezoelectric response, carrier transport dynamics, and photon emission mechanisms. In order to further promote this strategy, we codoped Mn^4+^ with RE ions into CaZnOS and utilized the self‐reduction of Mn^4+^ to synergistically boost the ML output of both species. As an example, the ML intensity in the NIR region for the Yb^3+`^ codoped system is enhanced by a factor of ≈3.3 compared to the singly doped counterpart. Furthermore, we developed a novel multilayer orthodontic sensor using CaZnOS: Nd^3+^ phosphors regulated by self‐reduction, capable of precisely detecting occlusal position and bite force magnitude. Coupled with neuromorphic computing and artificial neural network (ANN) training, the system achieved high‐accuracy ML image recognition with a 96.89% success rate in bite point localization. Additionally, the sensor demonstrated excellent linearity and repeatability in bite force detection. Ultimately, this self‐reduction‐enhanced orthodontic sensor shows great potential for applications in orthodontic assistance and human–machine interaction due to its superior multipoint recognition and high‐precision mechanical sensing capabilities.

## 2 Results and Discussion

### 2.1 Crystal Structure, Micromorphology, and Valence State Identification

**Figure 2:**
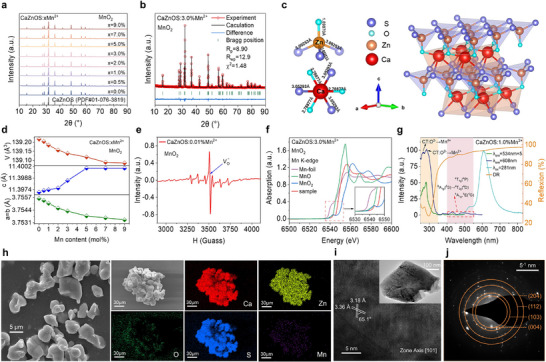
Crystal structure, Mn valence state, and surface morphology of self‐reduced samples. a) XRD patterns of CaZnOS: xMn^2+^ (*x* = 0, 0.5%, 1.0%, 2.0%, 3.0%, 5.0%, 7.0%, and 9.0%). b) Rietveld refinement and structural data for CaZnOS: 3.0%Mn^2+^. c) Crystal structure model of CaZnOS. d) Lattice parameters a,b,c) and unit lattice volume (V) as a function of Mn^2+^ concentration. e) EPR spectrum of CaZnOS: 0.01%Mn^2+^. f) Mn K‐edge XANES spectra of CaZnOS: 3.0%Mn^2+^, Mn foil, MnO, and MnO_2_; inset: enlarged pre‐edge region. g) PL spectrum (*λ*_ex_ = 281 nm), PLE spectra (*λ*_em_ = 534 and 608 nm), and DR spectrum of CaZnOS: 2.0%Mn^2+^. h) SEM (2000×) and corresponding EDS elemental mapping images of CaZnOS: 3.0%Mn^2+^. i) HRTEM image of CaZnOS: 3.0%Mn^2+^; inset: low‐magnification TEM image. j) SAED pattern of CaZnOS: 3.0%Mn^2+^.

A series of CaZnOS: xMn^2+^ (0.0% ≤ *x* ≤ 9.0%) phosphors were synthesized via a conventional high‐temperature solid‐state reaction method using MnO_2_ as the Mn source. For comparison, a control group of samples with identical Mn concentrations were prepared under the same conditions using MnCO_3_. X‐ray diffraction (XRD) patterns for both sets of samples match perfectly with the standard PDF card (JCPDS #01‐076‐3819), confirming that the incorporation of either Mn^2+^ or Mn^4+^ does not disrupt the host crystal structure or introduce impurities (**Figure** **2**a; and Figure S1a, Supporting Information). Additionally, Rietveld refinement yields reasonable R‐factors, further verifying the reliability of the structural fitting results (Figure 2b; and Figures S2 and S3a–g, Table S2, Supporting Information). The refined structure indicates that CaZnOS crystallizes in the noncentrosymmetric hexagonal space group P6_3_mc (No.186), comprising [ZnOS_3_] tetrahedra and [CaO_3_S_3_] octahedra. These distorted polyhedra, due to their lack of inversion symmetry, are prone to piezoelectric polarization under applied stress, laying a robust structural foundation for efficient ML (Figure 2c).^[^ ^24^ ^]^ Furthermore, the alternately stacked tetrahedral and octahedral layers along the *c*‐axis form a layered structure that facilitates efficient electronic transport. Considering the ionic radii and coordination environments of Ca^2+^ (*r* = 1.00 Å, CN = 6), Zn^2+^ (*r* = 0.60 Å, CN = 6), Mn^2+^ (*r* = 0.66 Å, CN = 4; *r* = 0.83 Å, CN = 6), and Mn^4+^ (*r* = 0.53 Å, CN = 6), and applying the Hume–Rothery rules (Equation (S1), Supporting Information), which state that the acceptable ionic radius mismatch should not exceed 30%, the calculated mismatch values Dr (Mn^2+^‐Ca^2+^) = 17% and Dr (Mn^2+^‐Zn^2+^) = 10% suggest that Mn^2+^ can feasibly substitute for both Ca^2+^ and Zn^2+^ site. As the Mn concentration increases, the lattice volume of MnCO_3_‐derived samples initially expands and then contracts, implying that Mn^2+^ preferentially substitutes for Zn^2+^ at lower concentrations (increasing the cell volume), followed by substitution at the larger Ca^2+^ sites (causing volume contraction) (Figure S1b, Supporting Information). In contrast, the samples synthesized from MnO_2_ show a consistent decrease in lattice volume with increasing Mn content. This could be due to the smaller ionic radius of Mn^4+^ and the cation vacancies introduced by its heterovalent substitution (Figure 2d).

To further confirm the spontaneous reduction of Mn^4+^ in the CaZnOS lattice, EPR spectroscopy was performed on an ultra‐low‐doped sample (*x* = 0.01%) prepared with MnO_2_. The EPR spectrum displays the characteristic six‐line hyperfine splitting of Mn^2+^, attributed to the interaction between unpaired electrons and the Mn nucleus (I = 5/2), confirming the presence of Mn^2+^ in the system (Figure 2e). Additionally, a strong signal near g = 2.003 indicates the presence of oxygen vacancies, suggesting that the reduction process is accompanied by defect formation (Figure S4, Supporting Information). XANES was conducted on the 3.0% MnO_2_‐doped sample and standard samples of different valence states. The absorption edge of the sample lies between those of MnO and MnO_2_, indicating the coexistence of Mn^2+^ and Mn^4+^ and confirming incomplete self‐reduction process (Figure 2f). In contrast, X‐ray photoelectron spectroscopy (XPS) of the MnCO_3_‐derived sample confirms the stable coexistence of Ca, Zn, O, S, and Mn in consistent oxidation states. The high‐resolution Mn 2p spectrum can be deconvoluted into two Gaussian peaks at 640.58 and 653.68 eV, aligning well with standard values for Mn^2+^ (2p_3/2_ = 641.7 eV, 2p_1/2_ = 653.5 eV).^[^ ^25^ ^]^ Additionally, the satellite peak at 646.68 eV, characteristic of Mn^2+^, further confirms the absence of valence change in reference samples (Figure S5, Supporting Information).

Subsequent microstructural characterization was conducted on the self‐reduced samples. Scanning electron microscopy (SEM) reveals irregular surface morphology with an average particle size of ≈5 µm. Energy‐dispersive X‐ray spectroscopy (EDS) mapping shows uniform distribution of Ca, Zn, O, S, and Mn across individual particles (Figure 2h). Transmission electron microscopy (TEM) images reveal lattice fringes with interplanar spacings of 3.18 and 3.36 Å, corresponding to the ($10\bar{1}$) and (010) lattice planes of CaZnOS, respectively, with an interplanar angle of 65.1° (Figure 2i). Selected area electron diffraction (SAED) patterns display polycrystalline diffraction rings with measured *d*‐spacings of *d*_1_ = 2.90 Å, *d*_2_ = 2.41 Å, *d*_3_ = 1.75 Å, and *d*_4_ = 1.41 Å, which are indexed to the (004), (103), (112), and (204) lattice planes of CaZnOS (Figure 2j).^[^ ^26^ ^]^

Under optimal ultraviolet (UV) excitation at 281 nm, both sets of CaZnOS: xMn^2+^ phosphors exhibit similar characteristics of photoluminescence (PL) spectra (Figure S6, Supporting Information). At lower Mn^2+^ doping levels, two distinct emission peaks are observed at 534 and 608 nm, designated as Mn (1) and Mn (2), respectively. However, with increasing Mn^2+^ concentration, the Mn (1) peak undergoes rapid and complete quenching process. To identify the origins of these two emission centers, crystal field splitting of Mn^2+^ in the CaZnOS lattice was analyzed using Tanabe–Sugano diagrams and ligand field theory (Figure S7, Supporting Information). By correlating the energy levels observed in the PLE and PL spectra (Figure 2g), the crystal field splitting parameter (Dq) was calculated (Equations (S2–S4), Supporting Information).^[^ ^26c^ ^]^ The calculation results show that the green emission at 534 nm is attributed to Mn^2+^ ions' occupying tetrahedral Zn^2+^ sites. Conversely, the orange emission at 608 nm corresponds to Mn^2+^ substituting into octahedral Ca^2+^ sites with stronger crystal fields. Both emissions result from the spin‐forbidden ^4^T_1_(^4^G) →^6^A_1_(^6^S) transition of Mn^2+^, with the energy difference governed by distinct ligand environments. The time‐resolved (TRES) PL curves further confirm that the two emission peaks belong to the same transition (Figure S8, Supporting Information). This dual‐emission behavior originates from the characteristic 3d^5^ electronic configuration of Mn^2+^ ions, whose emission properties are highly sensitive to the surrounding crystal field. Variations in crystal field strength, depending on the coordination environment, can lead to significant shifts in emission wavelength for the same electronic transitions.^[^ ^27^ ^]^

Compared to the control group, self‐reduced samples display slower and less pronounced concentration quenching effect, suggesting that the effective Mn^2+^ content in these samples is lower under identical nominal doping levels. This conclusion is further supported by fluorescence lifetime measurements (Figure S9, Supporting Information). Additionally, due to differences in the Mn (1)/Mn (2) intensity ratio and the quenching behavior between the two series, their PL color evolution show marked divergence. The self‐reduced samples exhibit slower migration of the CIE chromaticity coordinates (Figure S10a,b, Supporting Information), and their appearance under daylight and UV light is shown in Figure S10c–f (Supporting Information). To further explore the concentration quenching mechanism, the critical distance (*R*_c_) was calculated using the Blasse formula (Equation (S5), Supporting Information).^[^ ^17^, ^28^ ^]^ The result, being significantly greater than 5 Å, rules out exchange interactions as the primary quenching pathway. According to Dexter's energy transfer theory (Equation (S6), Supporting Information), linear fitting of log(I/x) versus log(x) yielded θ values of 5.25 and 5.43, indicating that the quenching process is predominantly governed by electric multipolar interactions and specifically dipole–dipole interactions between Mn^2+^ ions (Figure S11, Supporting Information).

The optical absorption behavior of the samples was evaluated using diffuse reflectance (DR) and PLE spectroscopy. As shown in Figure S12 (Supporting Information), both sets of samples exhibit the strongest excitation peak around 281 nm, attributed to the ligand‐to‐metal charge transfer (LMCT) from O^2−^ to Mn^2+^. It is worth noting that, in the self‐reduced samples, an additional excitation peak appears around 265 nm in the PLE spectra monitored at the two emission peaks. This feature corresponds to the LMCT band formed between residual Mn^4+^ ions and O^2−^ ligands, indicating that Mn^4+^ has been effectively incorporated into the lattice.^[^ ^23^, ^29^ ^]^ Furthermore, weak absorption features observed in the 420–530 nm range are assigned to the *d*–*d* transitions of Mn^2+^ (^6^A_1g_→^4^T_1g_, ^4^T_2__g_ and ^4^A_1g_), which correlate well with DR spectral features. The much higher intensity of the host excitation band compared to the Mn^2+^ absorption band confirms the presence of an efficient energy transfer mechanism from CaZnOS to Mn^2+^ dopants. DR analysis also reveals that the absorption associated with the Mn^4+^‐O^2−^ charge transfer band increases with doping level, indicating the accumulation of unreduced Mn^4+^ ions (Figure S13a, Supporting Information). Importantly, despite this absorption, Mn^4+^ does not contribute to any detectable photon emission in the system. Raman spectroscopy further elucidates structural changes induced by doping. The pristine CaZnOS lattice exhibits two E‐mode vibrations (symmetric E_1_ and antisymmetric E_2_) and one A_1_ mode. All doped samples retain the characteristic vibration peaks associated with [CaO_3_S_3_] octahedra (370 cm^−1^) and [ZnOS_3_] tetrahedra (540–630 cm^−1^) (Figure S14a, Supporting Information). However, as Zn^2+^ and Ca^2+^ sites are progressively substituted, the crystal symmetry is disrupted, leading to a systematic decrease in the intensity of the vibrational peaks.^[^ ^30^ ^]^ Due to the larger ionic radius, Mn^2+^ has a more pronounced impact on the Raman peak intensity. Comparative analysis shows that self‐reduced samples maintain stronger Raman signals than their control counterparts, providing direct structural evidence for incomplete Mn^4+^ reduction (Figure S14b,c, Supporting Information).

The optical bandgap (Eg) of the samples was estimated using the Kubelka–Munk function (Equations (S7–S8), Supporting Information). CaZnOS has an intrinsic bandgap of 4.22 eV, which decreases progressively to 3.76 eV as the Mn concentration increases (Figure S13b‐i, Supporting Information). This decline in bandgap is attributed to the Mn*─*O interactions introducing localized states within the bandgap, thereby reducing the energy required for electronic transitions.^[^ ^31^ ^]^ Finally, variable‐temperature emission spectroscopy demonstrated that the self‐reduced samples maintain stable Mn^2+^ emission even at elevated temperatures up to 500 K, confirming the excellent thermal stability of the self‐reduction process (Figure S15, Supporting Information).

### 2.2 Mechanoluminescence Properties of CaZnOS: Mn^2+^

**Figure 3:**
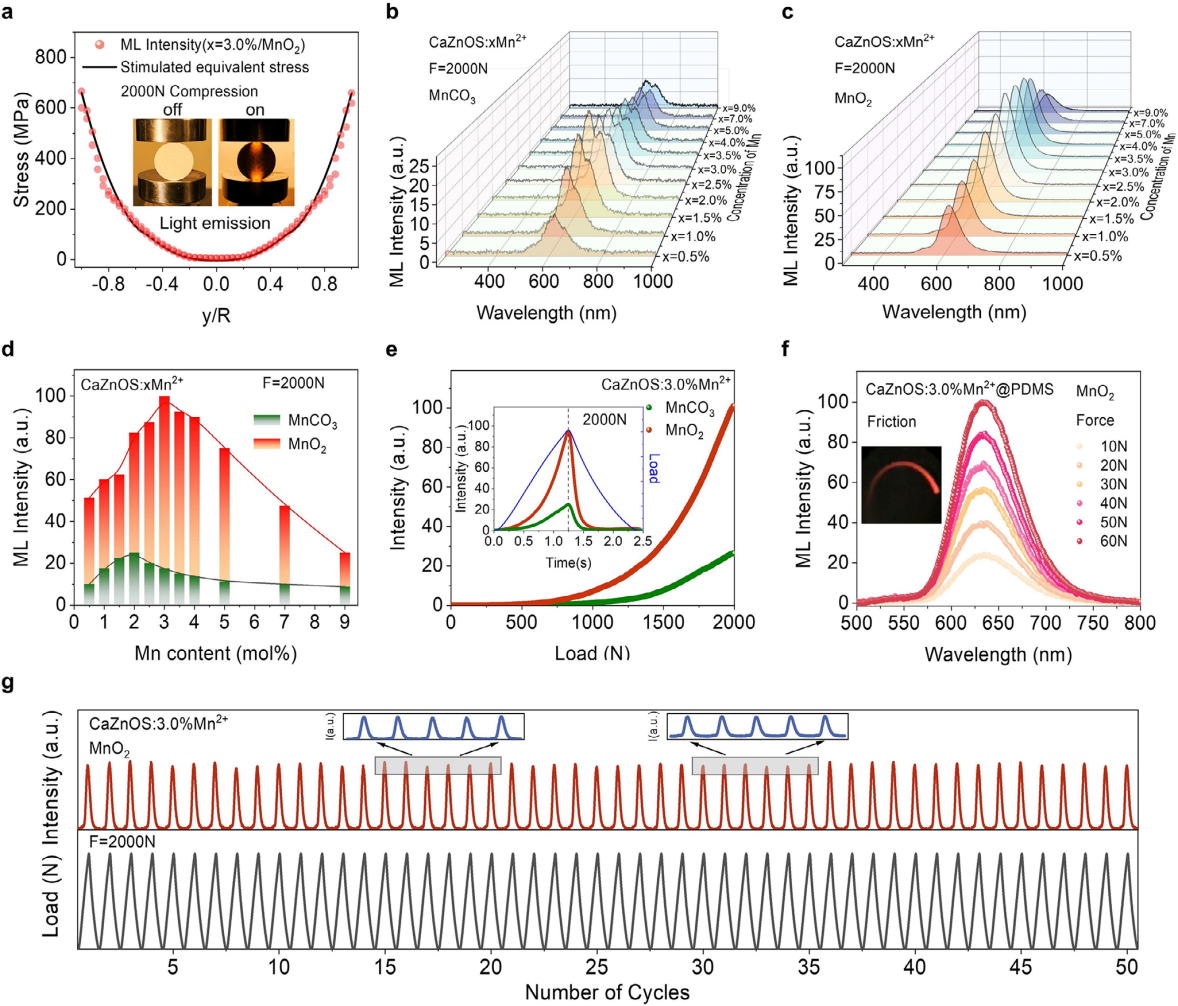
Significant improvement in ML performance by self‐reduction strategy. a) Comparison of ML intensity (along the vertical axis of CaZnOS: 3.0%Mn^2+^) at 2000 N pressure with simulated stress distribution. Inset: photographs of CaZnOS: 3.0%Mn^2+^ with self‐reduction under ambient light before (left) and during (right) compression. b,c) ML images of CaZnOS: xMn^2+^ (*x* = 0%–9.0%) under 2000 N uniaxial pressure b) with and c) without self‐reduction. d) ML peak intensity as a function of Mn^2+^ concentration with and without self‐reduction. e) ML intensity of CaZnOS: 3.0%Mn^2+^ with and without self‐reduction under 2000 N pressure. Inset: time‐dependent ML curves for both samples under triangular wave load. f) ML curves of CaZnOS: 3.0%Mn^2+^ under varying applied forces. g) ML stability and repeatability over 50 loading cycles at constant pressure.

To evaluate the influence of the self‐reduction strategy on the ML performance of CaZnOS: xMn^2+^, two sets of samples were embedded in epoxy resin and fabricated into cylindrical forms for ML testing. Finite element analysis was employed to simulate the stress distribution of a cylinder under a 2000 N load (Figure S16, Supporting Information). As shown in **Figure** **3**a, the stress profile along the vertical central axis (black line) was extracted, where *R* represents the cylinder radius and *y* denotes the vertical distance from the center.^[^ ^22^, ^32^ ^]^ Simultaneously, grayscale analysis of the ML emission images captured during loading was used to extract the ML intensity profile along the same axis (represented by red spheres). The near‐perfect overlap between the stress distribution and ML intensity profile confirms a strong linear correlation between applied stress and emitted light intensity. The inset of Figure 3a displays the ML response of the optimal self‐reduced sample before and during loading, in which intense ML emission can be clearly observed. Surprisingly, the ML spectrum of the control sample (synthesized using MnCO_3_) shows an additional emission peak in the deep red band which intensifies with increasing doping levels. Prior to this, Zhang et al. had already discovered that in CaZnOS: Mn^2+^, as the doping levels increases, the emission peak of Mn would undergoes a significant redshift, and they attributed this to the formation of Mn^2+^–Mn^2+^ pairs.^[^ ^33^ ^]^ Therefore, the additional emission peak is likely associated with dimer formation (Figure 3b). In comparison, the self‐reduced samples consistently exhibit only the characteristic emission peak of isolated Mn^2+^ at all doping levels (Figure 3c), suggesting that the self‐reduction process regulates the local distribution of Mn^2+^ within the host lattice. This hypothesis will be discussed in details later. A comparative analysis of ML intensities at identical doping levels confirms the performance enhancement introduced by the self‐reduction strategy. Obviously, self‐reduced samples exhibit significantly higher ML intensities. At various doping concentrations, the ML intensities of the self‐reduced samples are 3.0–6.7 times those of the reference samples, with the maximum ML emission intensity reaching up to 4 times that of the control samples (Figure 3d).

To further elucidate the dynamic relationship between applied load and photon emission, we quantified the number of photon emissions using an oscilloscope and a photomultiplier tube (PMT). As illustrated in Figure 3e, both samples show a monotonic increase in photon emission intensity with increasing mechanical load at *x* = 3.0%, verifying the positive correlation between stress and light output. Notably, the self‐reduced sample demonstrates a steeper increase in emission, indicating superior sensitivity to stress fluctuations and higher force‐to‐light conversion efficiency. The inset of Figure 3e presents the time‐dependent ML response of both samples under a 2000 N triangular wave load, visually demonstrating the enhancement in ML performance due to self‐reduction.

To assess the practical potential of this material for stress sensing applications, the self‐reduced phosphor (*x* = 3.0%) was embedded into a polydimethylsiloxane (PDMS) matrix and thermally cured to form flexible films for triboluminescence testing. The resulting ML spectra reveal a linear increase in emission intensity with applied pressure, achieving an excellent correlation coefficient of 99.03% (Figure 3f; and Figure S17, Supporting Information), confirming its outstanding pressure‐sensitive luminescent properties. Additionally, cyclic ML testing was performed on cylindrical samples under repeated 2000 N loading. After 50 load cycles, the samples retain nearly original ML intensity, demonstrating excellent self‐recoverability (Figure 3g). The above results highlight the promising application potential of self‐reduced ML materials in flexible mechanical sensors and other stress‐responsive luminescent devices.

### 2.3 Density Functional Theory Calculation to Assist in Confirming the Defects

The introduction of the self‐reduction strategy led to a remarkable enhancement in the ML intensity of CaZnOS: Mn^2+^, a result that is both exciting and significant. But it is crucial to deeply understand the underlying mechanism behind the enhancement of ML caused by self‐reduction. According to the principle of charge compensation in aliovalent substitution, the replacement of Ca^2+^ or Zn^2+^ by Mn^4+^ within the crystal lattice inevitably necessitates the formation of compensating defects to preserve charge neutrality. Existing studies have shown that during the sintering process of CaZnOS, side reactions are prone to occur,

(1) ZnS+CaO⇋CaZnOS⇋ZnO+CaS

(2)

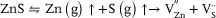



(3)

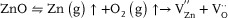

which make the generation of vacancies much easier than interstitials.^[^ ^6^, ^34^ ^]^ When Mn^4+^ undergoes heterovalent substitution, cation vacancies are formed to satisfy charge conservation, and the electrons release during the formation of anion vacancies may trigger the reduction reaction, driving the reduction of Mn^4+^ to Mn^2+^.

**Figure 4:**
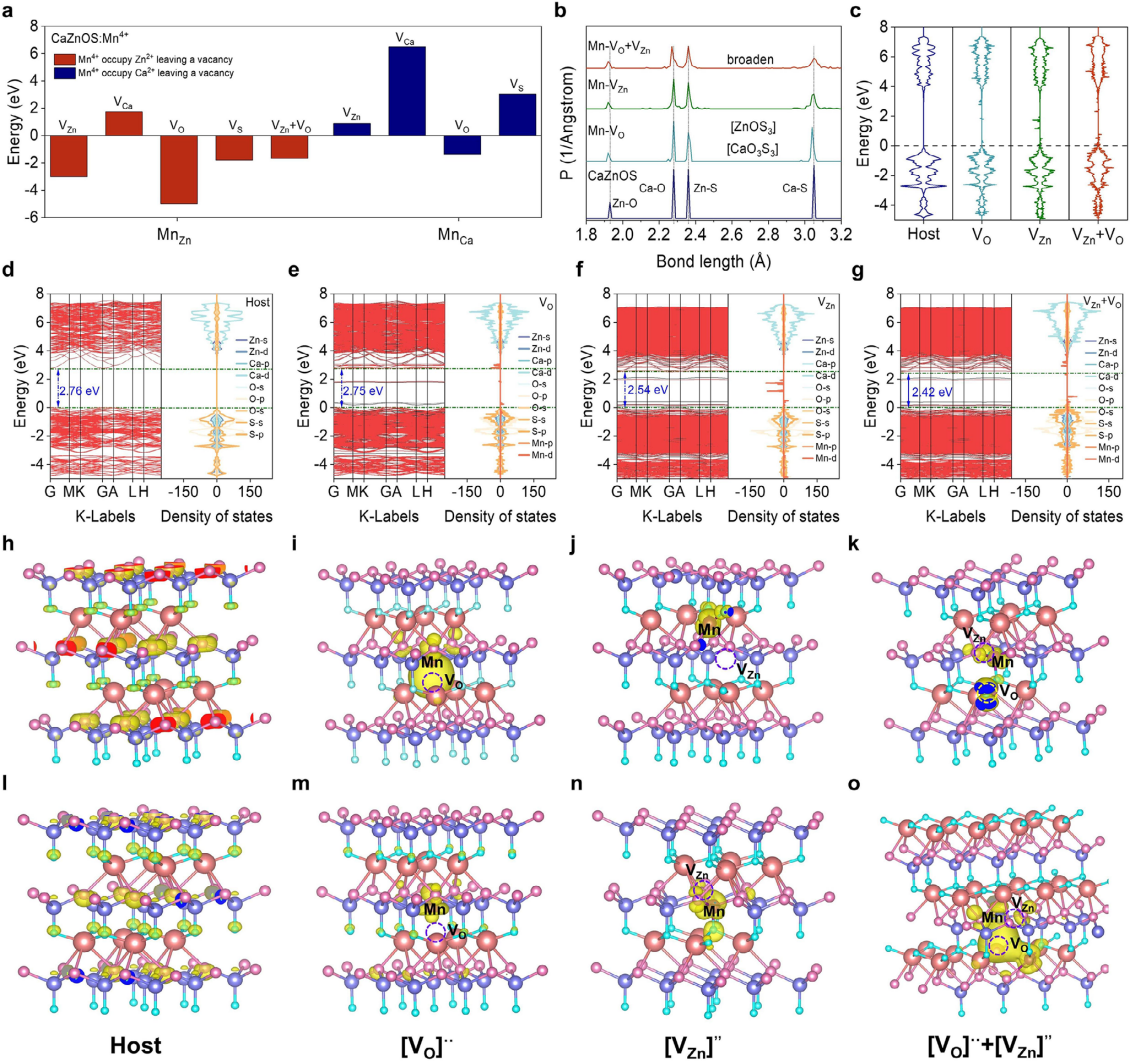
DFT calculation to confirm the defects introduced by self‐reduction. a) Formation energies of various defects induced by charge imbalance during self‐reduction. b) Bond length distributions under different model configurations. c) TDOS for four models; dashed lines indicate the Fermi level. d–g) Energy band structure and PDOS and Fermi energy level for d) pristine CaZnOS, e) CaZnOS: Mn^2+^ with $V_{O}^{\cdot \cdot }$, f) CaZnOS: Mn^2+^ with 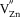, g) CaZnOS: Mn^2+^ with $V_{O}^{\cdot \cdot }$ and 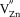. h–k) The charge density distribution diagrams of the CBM for the four models. l–o) The charge density distribution diagrams of the VBM for the four models.

To systematically investigate the types of defects introduced by the self‐reduction process and their correlation with ML performance, first‐principles calculations based on DFT were performed using the Vienna Ab initio Simulation Package (VASP). The electronic band structure and charge density distribution of CaZnOS were calculated. First of all, a 4 × 4 × 2 supercell (Ca_64_Zn_64_O_64_S_64_) was constructed, in which one Ca or Zn atom was replaced by Mn, along with the introduction of a vacancy to simulate the experimental conditions of Mn^4+^ self‐reduction. This configuration corresponds to an effective doping concentration of ≈1.5%. The calculated defect formation energy can be used to further verify the different defect formation process. The result reveals that configurations involving Mn substitution at Zn sites accompanied by either an oxygen vacancy ($V_{O}^{\cdot \cdot }$) or a zinc vacancy (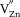) possess the lowest formation energies (**Figure** **4**a). Furthermore, the simultaneous presence of both $V_{O}^{\cdot \cdot }$ and 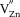 also results in a relatively low formation energy. All three defect scenarios exhibit negative formation energies, indicating structural stability and ruling out lattice collapse. Based on these findings, these three defect types, along with pristine CaZnOS, were selected for further comparative analysis.

To understand the influence of these defects on the local crystal field and structural distortion, bond length distributions were analyzed for each configuration (Figure 4b). After the introduction of Mn and vacancies, a broadening phenomenon of the bond length distribution is observed, indicating significant local structural distortion, which might be attributed to the Jahn–Teller effect. Under the coexistence of $V_{O}^{\cdot \cdot }$ and 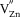, this distortion was particularly obvious, suggesting that the local crystal field perturbation was the greatest under this condition, and thus it might enhance the electron–phonon coupling effect and improve the ML efficiency. Figure 4c presents the total density of states (TDOS) for the four configurations, with the Fermi level indicated by the lower dotted line. Upon Mn incorporation, impurity states derived from Mn‐3d orbitals emerge within the bandgap. Electronic band structure and projected density of states (PDOS) (Figure 4d–g) analyses further reveal the energy level modulation mechanism. Pristine CaZnOS exhibits a direct bandgap value of 2.76 eV at the G point, which is smaller than the experimentally measured value. This discrepancy is attributed to the use of the Perdew–Burke–Ernzerhof (PBE) functional, a generalized gradient approximation (GGA) known to systematically underestimate bandgap values due to the omission of higher‐order electron correlation effects.^[^ ^35^ ^]^ Nonetheless, the qualitative trends remain valid. In all Mn‐doped and defective systems, a reduction in bandgap is observed. This is due to the presence of Mn‐3d states located near the mid‐gap region, facilitating electronic transitions from the valence band (VB) to the conduction band (CB) and enhancing radiative recombination. PDOS analysis indicates that the valence band maximum (VBM) primarily arises from O‐2p and S‐3p orbitals, while the conduction band minimum (CBM) is mainly composed of Zn‐4s and Ca‐3d orbitals. When both $V_{O}^{\cdot \cdot }$ and 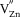 are introduced, Mn‐3d states concentrate near the Fermi level, resulting in the smallest calculated bandgap of 2.42 eV. This reduced bandgap is beneficial for charge excitation and storage, making the system more conducive to photon generation.

To further visualize the effect of defects on electronic structure, 2D charge density maps were generated along crystallographic planes intersecting the Mn dopants and defect sites. In these images, blue regions represent atomic nuclei, overlapping green and yellow regions depict valence electron clouds and their interactions, while red regions indicate low electron density or charge‐depleted zones. Comparison with pristine CaZnOS reveals the appearance of $V_{O}^{\cdot \cdot }$, 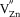, and $V_{O}^{\cdot \cdot }$ + 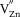 defects and clear changes in charge density around the defect sites (Figure S18, Supporting Information).

Analysis of the charge density distributions at the VBM and CBM for the four configurations reveals distinct behaviors (Figure 4h–o). In the presence of only $V_{O}^{\cdot \cdot }$, electrons tend to accumulate around the CBM. Conversely, when only 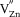 are introduced, 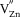 acts predominantly as a hole trap near the VBM. When both $V_{O}^{\cdot \cdot }\;$ and 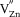 are present simultaneously, the electrons localized near the CBM (trapped by $V_{O}^{\cdot \cdot }$) and the holes near the VBM (trapped by 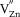) are in a closer spatial position, facilitating tunneling‐assisted carrier recombination. This process promotes the recombination of electron‐hole pairs, thereby activating the 3d–3d transitions of Mn^2+^ and enhancing the ML response.

Therefore, the self‐reduction process introduces $\left[V_{O}^{\cdot \cdot }+{V^{\prime \prime }}_{\mathrm{Zn}}\right]$ composite defects that effectively modulate local structural distortions, the electronic density of states, and the band structure. These defects play a decisive role in governing the excitation and radiative recombination pathways of electrons, thus providing a fundamental physical basis for the observed enhancement in ML performance. This insight offers a computational‐level understanding of the intrinsic mechanisms driving ML intensification in self‐reduced CaZnOS: Mn^2+^ systems.

### 2.4 ML Enhancement Mechanism Based on Self‐Reduction Strategy

**Figure 5:**
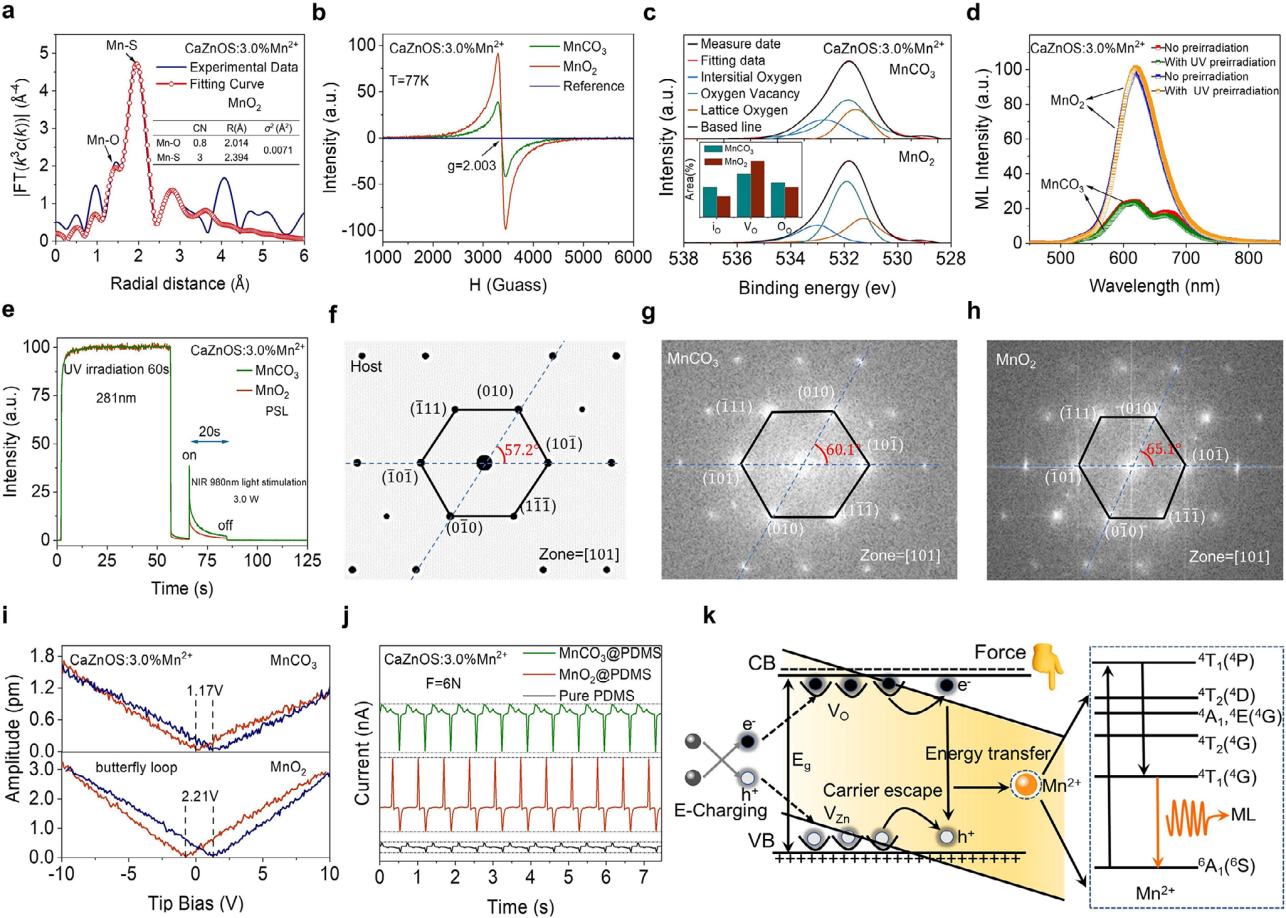
Mechanism analysis of enhanced ML performance. a) EXAFS experimental data and fitting curves in R‐space for CaZnOS: 3.0%Mn^2+^ with self‐reduction. b) Low‐temperature (77 K) EPR spectra of CaZnOS: 3.0%Mn^2+^ with and without self‐reduction versus control groups. c) O 1*s* XPS spectra of two samples. d) ML spectra of two samples before and after preirradiation. e) PSL spectra of CaZnOS: 3.0%Mn^2+^ with and without self‐reduction under 3.0 W, 980 nm laser irradiation for 20 s. f) Simulated electron diffraction pattern of ideal CaZnOS crystal viewed along the [101] zone axis. g,h) FFT diffraction patterns under the [101] zone axis obtained from CaZnOS: 3.0%Mn^2+^ particle with and without self‐reduction. i) PFM curves of two CaZnOS: 3.0%Mn^2+^ samples. j) Piezoelectric signals of CaZnOS: 3.0%Mn^2+^ with and without self‐reduction embedded in PDMS under 6 N cyclic loading, pure PDMS film was used as the control group k) Potential ML mechanism proposed based on the synergistic effect of the piezophotonic effect and defect regulation.

To experimentally validate the self‐reduction process, EXAFS spectroscopy and Fourier transform (FT) analysis were performed on the self‐reduced CaZnOS: 3.0%Mn^2+^ sample to investigate the local coordination environment of the reduced Mn^2+^ ions (**Figure** **5**a). Previous structural analysis indicates that Zn forms a tetrahedral coordination with one O and three S atoms, while Ca adopts an octahedral coordination with three O and three S atoms. It is expected that the reduced Mn^2+^ ions substitute both Zn^2+^ and Ca^2+^ cationic sites. EXAFS spectra reveal a prominent peak at 1.96 Å, corresponding to the nearest‐neighbor Mn‐S coordination. Fitting analysis shows an average Mn*─*S bond length of 2.394 Å with a coordination number of 3, consistent with Rietveld refinement results. Another peak at 1.47 Å is attributed to Mn*─*O coordination. The fitted Mn*─*O bond length is 2.014 Å, but the coordination number is only 0.8, significantly lower than the expected range of 1–3. This deviation suggests the presence of a substantial number of $V_{O}^{\cdot \cdot }$ in the local coordination environment of Mn^2+^, which also explains the elongated Mn*─*O bond. EPR spectroscopy is widely used for identifying the oxidation states of metal ions, and it is also an effective method for characterizing defects such as oxygen vacancies. EPR spectra of both sample series across various doping levels reveal that the characteristic six‐line hyperfine splitting of Mn^2+^ became progressively broadened with increasing Mn content, eventually forming a pair of symmetric Lorentzian signals near 3500 G (Figure S19, Supporting Information). Notably, at the same Mn doping level, the self‐reduced samples exhibit significantly stronger EPR signals. However, since the EPR signal for oxygen vacancies are overlapped with the broadened Mn^2+^ signal, low‐temperature (77 K) EPR measurements were performed to minimize Mn^2+^ signal interference. The EPR responses of self‐reduction and nonself‐reduction sample were tested at 77 K, with a 0.01% doped self‐reduction sample used as the control. The spectra demonstrate that the Mn^2+^ hyperfine signals completely vanish in the control sample, confirming that low‐temperature measurements effectively suppress Mn^2+^ interference. In contrast, the self‐reduced sample exhibits significantly stronger signal, providing direct evidence that the self‐reduction process promotes the formation of oxygen vacancies (Figure 5b). High‐resolution O 1*s* XPS spectra further confirm this effect. O 1*s* peak is deconvoluted into three components: lattice oxygen (530.7 eV), oxygen vacancies (531.3 eV), and interstitial oxygen (532.3 eV).^[^ ^15^, ^23^ ^]^ Comparative analysis reveals a significantly higher proportion of oxygen vacancies in the self‐reduced sample (Figure 5c). This supports a strong correlation between oxygen vacancy formation and the self‐reduction process. Additionally, inductively coupled plasma optical emission spectroscopy (ICP‐OES) of CaZnOS: 3.0%Mn^2+^ samples with and without self‐reduction reveal a notable decrease in Zn content in the self‐reduced sample, indicating a higher concentration of 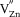 (Table S3, Supporting Information). These observational results are consistent with the computational results for the [$V_{O}^{\cdot \cdot }$+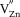] defect configuration. Based on both experimental and DFT results, the following self‐reduction reaction pathway is proposed.
(4)

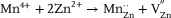

(5) $$O_{O}\to V_{O}^{\cdot \cdot }+\frac{1}{2}O_{2}+2e^{-}$$

(6) $${\mathrm{Mn}}_{\mathrm{Zn}}^{\cdot \cdot }+2e^{-}\to {\mathrm{Mn}}_{\mathrm{Zn}}^{\times \times \;}$$

Here, aliovalent substitution of Mn^4+^ introduces Zn^2+^ vacancies to maintain charge neutrality. The nitrogen atmosphere during synthesis creates an oxygen‐deficient environment, favoring the escape of lattice oxygen and the formation of oxygen vacancies. This process concurrently releases two electrons, which may quickly migrate through the covalent network of the layered CaZnOS structure and reduce Mn^4+^ to Mn^2+^ at designated sites. Moreover, as electrons released during oxygen vacancy formation are consumed in the reduction process, the equilibrium of the reaction shifts toward further vacancy formation, establishing a positive feedback mechanism. Notably, the simultaneous formation of a $V_{O}^{\cdot \cdot }$ and 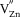 helps forming a full Mn^4+^ substitution and self‐reduction cycle.

The self‐reduction of Mn^4+^ in the CaZnOS lattice leads to the formation of stable composite defects involving $V_{O}^{\cdot \cdot }$+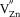 in the local coordination environment of Mn^2+^. These composite anion‐cation defects are identified as the direct physical origin of the significant ML enhancement observed in self‐reduced samples. To elucidate the role of self‐reduction‐induced defects in enhancing ML, we considered several widely accepted ML mechanisms in inorganic materials. On one hand, defects may act as carrier traps, increasing ML intensity by capturing more carriers. On the other hand, they may serve as structural lattice defects that induce local distortions, thereby enhancing the piezoelectric response and improving force‐to‐light conversion efficiency. To differentiate these contributions, comparative experiments were performed on self‐reduced and nonself‐reduced CaZnOS: xMn^2+^ samples with identical Mn doping concentrations.

In defect‐controlled ML mechanisms, medium‐depth traps (0.6–0.75 eV) are crucial for ML activation, as they store carriers that can be released upon mechanical stimulation. UV pre‐irradiation is often used to populate these traps and potentially restore ML performance. However, no significant differences are observed in the ML spectra of either sample before or after UV pre‐irradiation, indicating that UV photon‐charging does not influence their ML behavior (Figure 5d). Thermoluminescence (TL) measurements show a substantial increase in intensity upon Mn doping, confirming the generation of defects. However, the self‐reduced samples exhibit markedly lower TL signal, showing self‐reduction has led to a decrease in the trap concentration (Figure S20a, Supporting Information). Additionally, photostimulated luminescence (PSL) measurements, which is commonly used to probe carrier trap concentrations, were also performed. PSL refers to the re‐emission of stored energy in the form of light upon stimulation by external radiation, such as a laser. Figure S20b (Supporting Information) suggests that under 980 nm laser excitation, carriers trapped in deeper energy states are gradually pumped into shallower traps, from which they are subsequently released. During phosphorescence decay, excitation of 980 nm laser results in the transfer of carriers from deep to shallow traps, causing a sudden increase in emission intensity. Both samples were precharged under UV for 1 min, allowed to decay for 10 s, and then stimulated using a 3.0 W 980 nm laser.^[^ ^12b^ ^]^ Both exhibit PSL behavior; however, the self‐reduced sample displayed significantly weaker PSL intensity, further ruling out enhanced carrier trap concentration as the source of improved ML performance (Figure 5e). Given that both samples were embedded in the same matrix, differences in ML performance are unlikely to arise from interfacial charge transfer at the particle‐matrix boundary.

Considering the intrinsic piezoelectric nature of CaZnOS, it is plausible that the self‐reduction process enhances local lattice distortions, which in turn boosts the material's piezoelectric response and ML performance. Piezoelectricity is closely linked to the noncentrosymmetric nature of the crystal structure, and the degree of structural distortion is a critical factor influencing the piezoelectric response. To verify this hypothesis, simulated electron diffraction patterns were generated for ideal CaZnOS crystals along the [101] zone axis (Figure 5f). This is compared with fast Fourier transform (FFT) diffraction patterns obtained experimentally from CaZnOS: 3.0%Mn^2+^ particles with and without self‐reduction (Figure 5g,h). Special attention was given to the hexagonal symmetry formed by the six crystallographic directions: (010), ($10\bar{1}$), ($1\bar{1}\bar{1}$), ($0\bar{1}0$), ($\bar{1}0\bar{1}$), and ($\bar{1}1\bar{1}$). The comparison reveals clear geometric distortions in the self‐reduced sample, indicating significant structural reconstruction as a result of the self‐reduction process. PFM measurements further confirm enhanced piezoelectricity in the self‐reduced sample, showing a larger amplitude response and increased butterfly loop area under identical applied voltages (Figure 5i). This demonstrates that the self‐reduced sample can generate stronger internal polarization fields under identical mechanical stress, thereby facilitating more efficient charge separation and recombination, and ultimately improving ML efficiency. Furthermore, both types of samples were embedded in flexible PDMS and PVDF substrates to form composite films. Under periodic mechanical impacts, these films were assessed for their electrical signal response. The results show that compared with pure substrates, CaZnOS‐containing composites exhibit significantly enhanced piezoelectric voltage and current outputs, especially in the self‐reduced films, which deliver much higher responses than their nonself‐reduced counterparts, highlighting their superior piezoelectric properties (Figure 5h; and Figure S21, Supporting Information)

In summary, the schematic diagram of the ML mechanism is shown in Figure 5k, the self‐reduction process introduces coupled anion–cation defects ($V_{O}^{\cdot \cdot }$+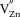) into the noncentrosymmetric hexagonal CaZnOS matrix, inducing lattice distortions and significantly enhancing its piezoelectric response. Under applied mechanical stress, the enhanced polarization field promotes more effective separation of charge carriers. Among them, electrons are captured by $V_{O}^{\cdot \cdot }$‐related traps close to the CBM, while holes are captured by 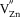
‐related traps near the VBM. Under a stronger internal polarization electric field, the valence band and conduction band exhibit more severe tilting, making the captured electrons easier to escape and enter the conduction band, and then through the tunneling effect, recombine with the holes near the VBM and transfer the energy to the luminescence center in a nonradiative form. This greatly increases and enhances the rate of carrier separation and recombination, ultimately significantly increasing the intensity of the generated ML (Movies S1 and S2, Supporting Information).

### 2.5 Optimization of the Local Distribution of Mn^2+^ by Self‐Reduction

**Figure 6:**
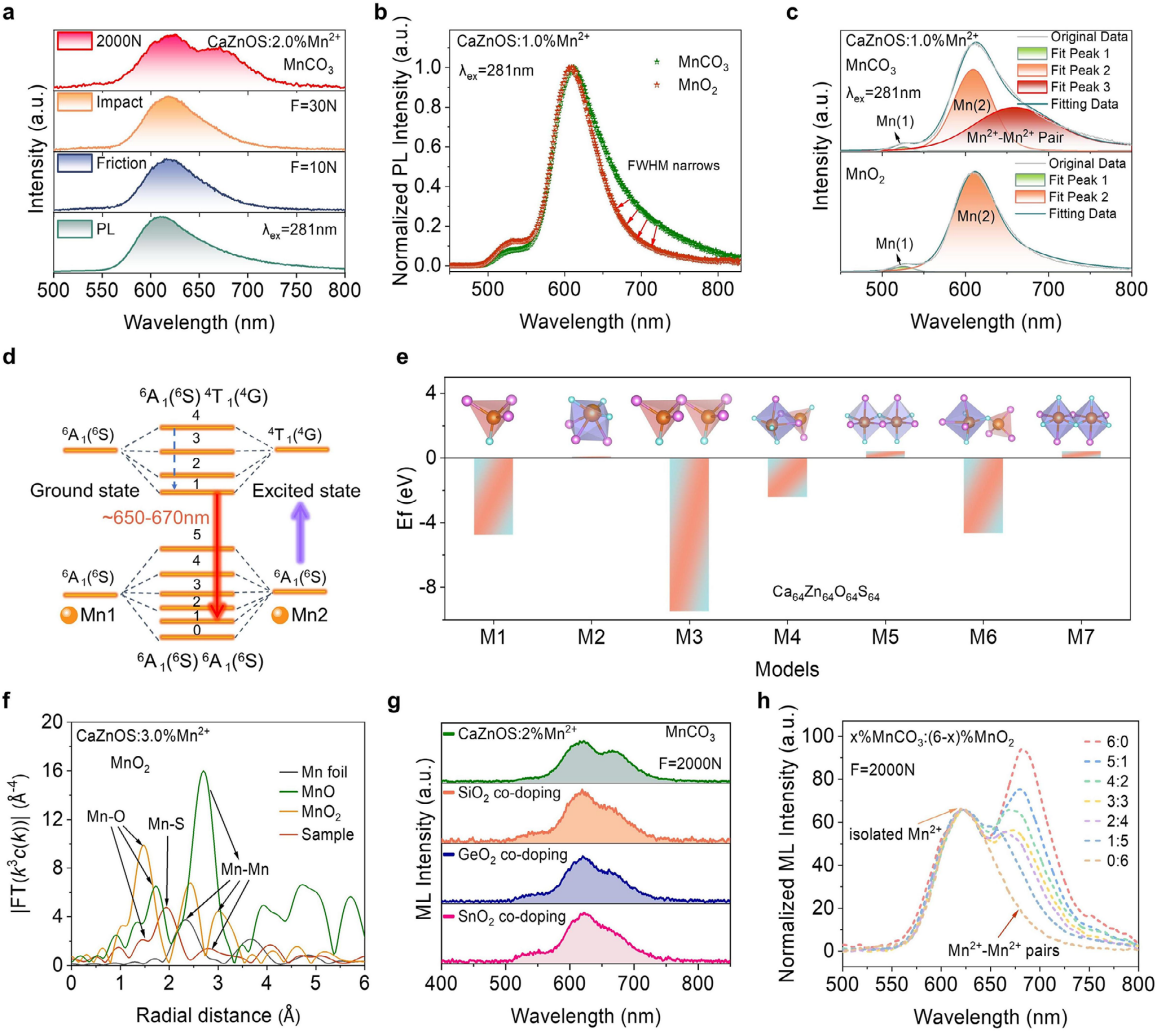
Local optimization of Mn^2+^ by self‐reduction. a) Luminescence spectra of CaZnOS: 2.0%Mn^2+^ without self‐reduction under various excitations. b) Normalized ML spectra of CaZnOS: 1.0%Mn^2+^ with and without self‐reduction under 281 nm excitation. c) Peak‐fitting analysis of PL spectra (at 534, 608, 650 nm) for two CaZnOS: 1.0%Mn^2+^ samples. d) Schematic of Mn^2+^ pair‐induced luminescent mechanism. e) Formation energies for different Mn substitution models (M1‐M2: single Mn; M3‐M7: dual Mn). f) EXAFS data and fits in R‐space for CaZnOS: 3.0%Mn^2+^, Mn foil, MnO, and MnO_2_, with peak assignments. g) ML spectra of codoped CaZnOS: 2.0%Mn^2+^ without self‐reduction (with Si^4+^, Ge^4+^, Sn^4+^). h) Normalized ML images (608 nm emission) of CaZnOS: 6.0%Mn^2+^ with varying Mn source ratios under 2000 N pressure.

In previous discussion, we observed that the CaZnOS: xMn^2+^ samples without self‐reduction exhibited an additional emission peak in the deep red region of their ML spectra under a high external load of 2000 N, particularly at higher doping concentrations. To identify the origin of this additional emission, we compared the PL and ML behaviors of a CaZnOS: 2.0%Mn^2+^ sample without self‐reduction under various excitation conditions. As shown in **Figure** **6**a, the emission spectra under 281 nm light excitation, mechanical impact, and friction all display notable asymmetry and long‐wavelength tails. Under compressive stress of 2000 N, however, a distinct new emission peak emerges around 660 nm in the ML spectrum. Further comparison of the normalized PL spectra of CaZnOS: 1.0%Mn^2+^ with and without self‐reduction reveals better spectral symmetry and a narrower full width at half maximum (FWHM) for self‐reduced samples (Figure 6b). Peak‐fitting analysis of the PL spectra show that the emission peaks of the samples without self‐reduction could be decomposed into three distinct peaks at 534, 608, and 650 nm (Figure S22 and Table S4, Supporting Information). The intensity contribution of the 650 nm peak, which is associated with Mn^2+^‐Mn^2+^ dimer formation, increases with higher Mn^2+^ concentrations (Figure S23, Supporting Information). This suggests that in singly Mn‐doped CaZnOS systems, three distinct emission centers may coexist. In contrast, the self‐reduced samples consistently exhibit only two emission peaks at 534 and 608 nm, indicative of isolated Mn^2+^ emission (Figure 6c).

Considering the properties of transition metal ions, Mn^2+^ tends to transition from isolated states to aggregated clusters as the doping concentration increases in specific matrices, ultimately forming Mn^2+^‐Mn^2+^ dimers that exhibit broad emission profiles.^[^ ^36^ ^]^ For isolated Mn^2+^ ions, the transition from the ground state (^6^A_1_, S = 5/2) to the lowest excited state (^4^T_1_, S = 3/2) is spin‐forbidden (ΔS = 1), resulting in a long fluorescence lifetime in the millisecond range. However, when Mn^2+^ forms Mn^2+^‐Mn^2+^ dimers via magnetic interaction, their spin coupling mechanism undergoes a fundamental transformation. In the dimeric state, the total spin quantum number (S) of the ground state (^6^A_1_(^6^S) ^6^A_1_(^6^S)) splits into five components (S = 5, 4, 3, 2, 1), while the excited state (^6^A_1_(^6^S)^4^T_1_(^4^G)) generates four components (S = 4, 3, 2, 1). This spin‐state reconstruction allows transitions with ΔS = 0 to become spin‐allowed (Figure 6d). Therefore, the magnetic interaction between Mn^2+^ and Mn^2+^ can alter the spin configuration of electrons and break the spin selection rule of *d*–*d* transition in Mn^2+^. forming a new emission center in the deep‐red spectral region.

DFT simulations were performed to investigate Mn^2+^ site preference and Mn^2+^‐Mn^2+^ dimer formation in CaZnOS across varying doping concentrations (Figure 6e). A 4 × 4× 2 supercell (Ca_64_Zn_64_O_64_S_64_) was constructed to evaluate both single and dual Mn^2+^ substitution models. The formation energy (*E*_f_) for Mn^2+^ occupying Zn^2+^ (M1, Mn/Zn) and Ca^2+^ (M2, Mn/Ca) sites was calculated, revealing a significantly lower Ef for M1 (−4.76 eV) compared to M2 (0.16 eV). This indicates that Mn^2+^ preferentially replaces Zn^2+^ at low doping concentrations, in agreement with Rietveld refinement results. Moreover, in this crystal structure, the formation energies for Zn and Ca substitutions at different lattice sites are identical, respectively. This indicates that all the positions occupied by Zn (Ca) within the lattice are energetically equivalent (Table S5, Supporting Information). To explore Mn^2+^‐Mn^2+^ dimer formation, two adjacent Mn^2+^ ions were introduced into the supercell, and all possible configurations were assessed. Given the substantial size mismatch between Ca^2+^ and Zn^2+^ (exceeding 30%), cross‐site substitution is improbable. Furthermore, Mn^2+^‐Mn^2+^ dimerization requires a shared anionic linkage. Under these constraints, five substitutional models were identified: For S‐bridged configurations, three models were identified: Mn/Zn|Mn/Zn (M3, both Mn^2+^ ions occupy adjacent Zn^2+^ sites), Mn/Zn|Mn/Ca (M4, one Mn^2+^ substitutes Zn^2+^, while the other replaces an adjacent Ca^2+^), and Mn/Ca|Mn/Ca (M5, dual Mn^2+^ occupying adjacent Ca^2+^ sites). O‐bridged configurations yielded two models: Mn/Zn|Mn/Ca (M6, one Mn^2+^ occupies the Zn^2+^ site, whereas the second Mn^2+^ ion substitutes at a neighboring Ca^2+^ position) and Mn/Ca|Mn/Ca (M7, structurally equivalent to M5). It should be emphasized that M5 and M7 represent different manifestations of the same structural configuration. Among these, M3 exhibits the lowest formation energy (−9.49 eV), suggesting that Mn^2+^ preferentially occupy two adjacent Zn^2+^ sites, leading to the formation of dimers. The calculated Mn^2+^‐Mn^2+^ interatomic distance in this configuration is 3.75 Å, reinforcing the feasibility of dimer formation. The relatively low formation energies of M4 (−2.42 eV) and M6 (−4.67 eV) suggest that as Mn^2+^ doping increases, substitution at Ca^2+^ sites become more favorable, explaining the origins of the Mn (1) and Mn (2) emission peaks observed in PL spectra.

Here, it can be inferred that this additional emission peak in the samples without self‐reduction is attributed to the formation of Mn^2+^–Mn^2+^ dimers in the CaZnOS matrix. Due to the uneven distribution of Mn^2+^ ions within CaZnOS, multiple emission are observed from isolated Mn^2+^ and aggregated Mn^2+^–Mn^2+^ pairs. Moreover, unlike under UV irradiation, friction, or impact, where the emission spectra only show significant asymmetry and broadening changes, the material undergoes distortion and the distance between Mn^2+^ ions becomes closer under high pressure may be the reason why the emission peak of the Mn^2+^‐Mn^2+^ pair becomes more prominent at a load of 2000N.

Experimental results show that effective suppression of Mn^2+^‐Mn^2+^ dimer formation in CaZnOS: Mn^2+^ samples with self‐reduction. To investigate the local structure around Mn, EXAFS measurements were performed. As shown in Figure 6f, the Fourier‐transformed EXAFS spectrum of the self‐reduced CaZnOS: 3.0%Mn^2+^ sample is compared with those of Mn foil, MnO, and MnO_2_ standards (Figure 6f; and Figure S24, Supporting Information). At the same time, further classification was carried out for each peak. The self‐reduced sample exhibits two primary coordination peaks, which are assigned to Mn‐O and Mn‐S interactions, respectively. Notably, its spectrum shows very weak signals within the range of 2.5–3.0 A, and these features are typically associated with the Mn–Mn scattering paths observed in MnO and Mn foils, which indicates that the Mn^2+^ ions mainly exist in isolated state rather than forming dimers or clusters.^[^ ^23^, ^35^ ^]^

Given that Mn^2+^ dimer formation requires an interionic distance below 5 Å and a shared anion bridge, we hypothesized that unreduced Mn^4+^ ions serve as spatial separators, effectively preventing Mn^2+^‐Mn^2+^ magnetic interaction. To verify this hypothesis, we codoped nonluminescent tetravalent ions (Si^4+^, Ge^4+^, Sn^4+^) into nonself‐reduced CaZnOS: 2.0%Mn^2+^ samples to simulate the spatial separation effect. The results show that PL spectra exhibit significantly reduced FWHM, and the deep‐red ML emission under 2000 N is effectively suppressed, confirming that this strategy substantially limits Mn^2+^‐Mn^2+^ dimer formation (Figure 6g; and Figure S25, Supporting Information).

To further confirm the suppressive effect of Mn^4+^ on the formation of Mn^2+^‐Mn^2+^ dimers, we synthesized a series of CaZnOS: Mn^2+^ and maintained the total Mn concentration at *x* = 6.0% while varying the Mn^2+^/Mn^4+^ ratio. Then the ML spectra under 2000 N were recorded and the spectra were normalized with respect to the isolated Mn^2+^ emission peak at 608 nm (Figure 6h). The normalized ML spectra clearly show that as the proportion of MnO_2_ increases, the relative intensity of the dimer‐related emission steadily decreases and eventually disappears when the ratio of MnO_2_ reaches 100%, leaving only the emission from isolated Mn^2+^ ions. This strongly supports the role of Mn^4+^ in regulating the local distribution of Mn^2+^. Interestingly, both PL and ML intensities increase with higher MnO_2_ ratios. This trend is attributed to the decreased actual concentration of Mn^2+^ ions due to incomplete self‐reduction of Mn^4+^, which suppresses nonradiative quenching (Figure S26, Supporting Information). A series of phosphors with 1% Mn doping were synthesized using MnCO_3_, Mn_2_O_3_, MnO_2_, and KMnO_4_ as Mn sources. All samples exhibit characteristic Mn^2+^ emission, demonstrating the excellent self‐reduction capability of the CaZnOS host (Figure S27, Supporting Information). Moreover, the results further confirm the effectiveness of spatial separation in suppressing the formation of Mn^2+^–Mn^2+^ pairs.

Under identical excitation conditions, the presence of multiple emission centers causes energy dispersion, which significantly limits emission efficiency. The self‐reduction strategy effectively suppresses PL and ML emissions from Mn^2+^‐Mn^2+^ dimers, concentrates energy on isolated Mn^2+^ centers, and eliminates energy loss due to magnetic interactions. This spectral simplification clearly contributes to enhanced luminescence output. A comparison of PL peak intensities at various Mn^2+^ concentrations confirms that self‐reduced samples exhibit higher PL intensity (Figure S28, Supporting Information). Therefore, the substantial enhancement in ML observed in self‐reduced CaZnOS: xMn^2+^ samples is partly due to optimized Mn^2+^ spatial distribution. Compared with the moderate increase in PL intensity, the dramatic improvement in ML intensity is primarily driven by enhanced piezoelectricity arising from self‐reduction‐induced anion‐cation defect complexes, which significantly improve the force‐to‐light conversion efficiency.

### 2.6 Promotion of Self‐Reduction Strategy and Application in Intelligent Sensing Devices

**Figure 7:**
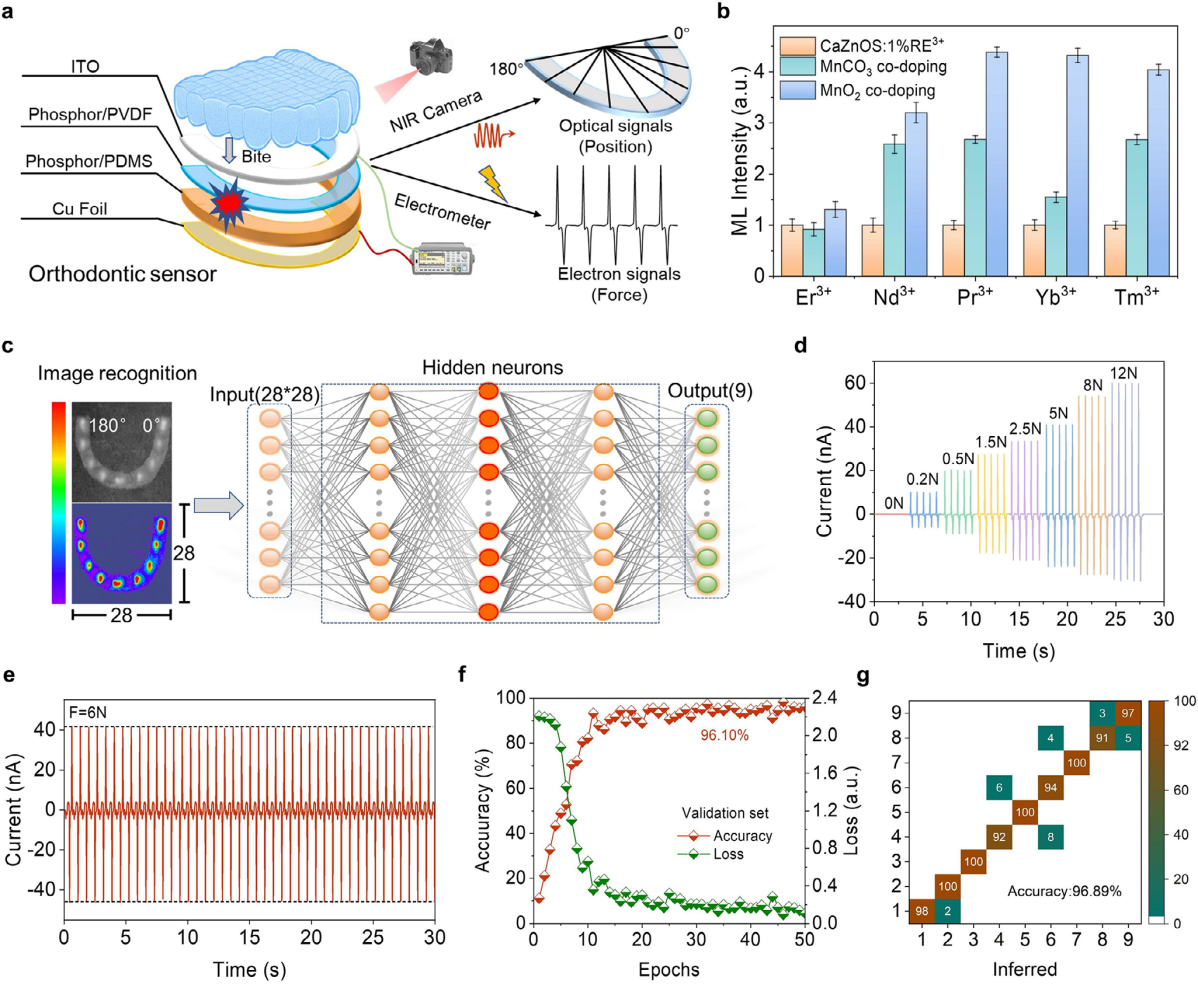
Further promotion of self‐reduction enhanced ML strategy and potential application. a) Schematic of an orthodontic appliance with dual optoelectrical output for detecting occlusal position and force magnitude. b) Enhancement ratio of near‐infrared ML via self‐reduction in RE ions codoped systems. c) ML images at various occlusal positions and schematic of the ANN architecture. d) Current signals output under different occlusal forces (0–12 N). e) Stability and repeatability of current signals over multiple occlusal cycles. f) ANN recognition accuracy for occlusal positions versus training epoch count in the validation set. g) Confusion matrix for 9 occlusal region classifications.

Based on the above analysis, we confirmed that the introduction of high‐valent dopant ions into the CaZnOS matrix through a self‐reduction strategy can induce the formation of composite defects and significantly enhance the degree of lattice distortion, thereby improving the piezoelectric properties of the material and enhancing its ML intensity. Based on this principle, with the help of the self‐reduction process of Mn^4+^ in CaZnOS, we achieved a dual improvement in the ML intensity and piezoelectric output of CaZnOS: Mn^2+^, significantly expanding its application potential in mechanical response sensors. In this study, a new type of orthodontic sensor was developed, which can accurately measure the bite position and bite force based on the coordinated detection of photoelectric signals.^[^ ^37^ ^]^ The sensor adopts a multilayer structure design: the top layer is a transparent ITO electrode, the bottom layer is a Cu foil electrode, and the middle layer is a double‐layer functional layer composed of a composite of phosphor and an elastic polymer (**Figure** **7**a). The side close to the ITO electrode is the light‐emitting layer, which is responsible for the output of the ML signal. And the other side close to the Cu foil is the electricity generation layer, which mainly responds to the piezoelectric effect caused by the bite force. This structure not only ensures the effective transmission of the piezoelectric signal to the electrometer for recording, but also ensures that the transparent electrode above will not block the collection of the ML light signal, thereby improving the camera capture efficiency (Figure S29, Supporting Information). It is worth mentioning that although the ML intensity can also be used to estimate the bite force, substantial errors may occur due to the different tissue thicknesses that various luminescent units must penetrate when reaching the camera. The piezoelectric signal can effectively circumvent this problem and provide a more accurate basis for the quantification of the bite force. The enhanced ML intensity brought about by the self‐reduction strategy has significantly optimized the signal‐to‐noise ratio and identification accuracy of the orthodontic sensor. At the same time, the enhancement of the piezoelectric signal has greatly improved the precision of bite force detection. However, the ML emission band of Mn^2+^ is mainly concentrated in the visible light region of 500–800 nm, and its penetration rate into human tissue is low, which limits its expansion in biological applications. At present, the types of ions with self‐reduction potential are relatively limited, including only Mn^4+^, Eu^3+^, Ce^4+^, and Bi^3+^, which greatly restricts the band range of self‐reduction regulation. Therefore, it is imperative to promote the self‐reduction control strategy and expand the control range of this strategy.

In order to break through this bottleneck, this study innovatively proposed a RE ions and Mn^4+^ codoping strategy, in which Mn^4+^ causes lattice distortion through the self‐reduction process, improves the piezoelectric response ability of the material, and thus achieves the synergistic enhancement of the RE ion ML performance. In the experimental design, a codoping system with a fixed Mn^4+^ doping amount (2 mol%) and a RE ion doping amount (1 mol%) was used, and a single‐doped RE ion system and a MnCO_3_/RE codoping system were set as control groups to exclude the influence of energy transfer between Mn^2+^ and RE ions on ML intensity, and then one can obtain a real and reliable ML intensity enhancement ratio. The results show that the self‐reduction strategy shows a consistent enhancement effect on all tested RE ions, and it achieves a significant ML improvement on the basis of the two control groups. Since the broadband emission of Mn^2+^ (500–800 nm) overlaps spectrally with the visible emission band of most RE ions (such as Eu^3+^ and Tb^3+^), it is difficult to accurately distinguish whether the ML enhancement originates from Mn^2+^ or the co‐doped RE ions. (Figure S30, Supporting Information). Therefore, in order to distinguish from the emission band, this study focuses on selecting ions with NIR characteristic emission such as Nd^3+^, Yb^3+^, Tm^3+^, Er^3+^, Pr^3+^, and accurately evaluates the ML enhancement effect by monitoring their NIR emission band. The experimental results show that the three‐step cascade mechanism of “Mn^4+^→Mn^2+^ valence state transition‐lattice distortion‐piezoelectric enhancement” induced by self‐reduction greatly improves the conversion efficiency of force to light signals. For example, the ML intensity of the Yb^3+^ codoped system in the NIR region is about 3.3 times higher than that of the single‐doped system (Figure 7b; and Figure S31, Supporting Information). A detailed comparison of the ML performance of our self‐reduced material with other representative systems was shown in Figure S32 (Supporting Information). Compared with other high‐performance ML materials reported in the literature, our self‐reduced CaZnOS‐based phosphors demonstrate excellent performance. Considering that Nd^3+^ co‐doped material exhibits the best ML performance in the NIR region, we further applied CaZnOS: Nd^3+^ regulated by self‐reduction to the orthodontic sensor to realize the bite position detection under NIR ML imaging. In terms of elastomer packaging materials, we encapsulated the CaZnOS: Nd^3+^ phosphor in PDMS, OE, Ecoflex and PVDF to make film materials, and unified the thickness and size. After comprehensively considering the piezoelectric output, ML intensity, mechanical properties and cycle stability, PVDF was finally selected as the light‐emitting layer matrix and PDMS as the electricity generation layer matrix (Figure S33, Supporting Information). To verify the tissue penetration ability of CaZnOS: Nd^3+^ regulated by self‐reduction, the transmittance of the sample covered by biological tissues of different thicknesses was tested. Although Nd^3+^ doped one has the strongest emission at 1091 nm, we selected the 913 nm wavelength as the detection index due to the decreased sensitivity of the spectrometer in this band (Figure S34, Supporting Information). The test shows that the self‐reduction strategy significantly increases the tissue penetration depth of CaZnOS: Nd^3+^ from 12 to 24 mm, which fully meet the application requirements of the orthodontic sensor (Figure S35, Supporting Information). Considering the bite habit of people, the braces are divided into 9 areas. The luminous points of the ML images taken will change according to the different bite areas. However, due to the low resistance of Cu foil and ITO electrodes, the piezoelectric signal measured by the electrometer is almost not affected by the bite position (Figure S36, Supporting Information).

In order to realize the image recognition and position classification of the luminous area of the orthodontic sensor, we introduced an ANN for neuromorphic computing. The network is trained by supervised learning and is used to classify and identify the ML images caused by different bite areas, so as to achieve high‐precision determination of the bite position of the brace wearer. We first established a training set of 720 sample images and an independent test set of 180 images. Each image is processed to be a 28 × 28‐pixel grayscale image, which comes from the standardization and grayscale normalization of the ML intensity images of different light‐emitting units on the sensor (Figure 7c). The pixel value of the image is directly converted into an input vector, reflecting the change of the ML intensity of each pixel. The neural network contains an input layer with 784 neurons, corresponding to 28 × 28 pixels. The three hidden layers provide stronger expression ability, can capture more complex spatial patterns and nonlinear features in ML images, and have more outstanding ability to distinguish similar areas. The output layer consists of 9 neurons, corresponding to the 9 independent luminous areas divided by the orthodontic sensor. The training results show that the neural network model achieves a recognition accuracy of 98.01% (Figure S37, Supporting Information) on the training set and maintains a high level of accuracy of 96.10% on the validation set, demonstrating excellent learning and generalization capabilities (Figure 7f). In addition, by plotting the confusion matrix to visualize the classification performance of the network. In the evaluation on the independent test set, the overall recognition accuracy of the model reaches 96.89% (Figure 7g). It can be clearly observed that all correct classifications are concentrated on the diagonal, indicating that the model has a high ability to distinguish different luminous areas. Only a very small number of samples have regional recognition misjudgments, and they are mainly concentrated in areas with weak light intensity or interference between adjacent luminous units, showing the superiority of the model in identifying the position of luminous units. In addition, the piezoelectric signal and the bite force show a good linear response relationship. In the range of 0–12 N, the output current increases steadily from 0 to 60 nA, and there is a one‐to‐one correspondence (Figure 7d). The repeatability test shows that after 40 cycles of testing under 6 N, the signal intensity does not change significantly, verifying the excellent stability of the device (Figure 7e). In summary, the self‐reduction enhanced orthodontic sensor shows broad application prospects in multipoint recognition and high‐precision mechanical testing.

## 3 Conclusion

In summary, this study establishes a facile and generalizable strategy based on self‐reduction to enhance the ML performance of CaZnOS‐based materials. By exploiting the spontaneous reduction process of Mn^4+^ to Mn^2+^, this approach generates defect complexes and induces significant lattice distortion that synergistically strengthen the internal piezoelectric response, thereby markedly increasing ML intensity. Comprehensive experimental and theoretical analyses elucidate the defect‐dominated mechanisms underlying the performance enhancement. Furthermore, the strategy proves versatile method for effectively improving NIR ML emission in RE codoped systems. Further work should be focused on regulating the self‐reduction process throughstrategies such as carbon paper encapsulation and reducing atmosphere sintering, and ultimately further improve the ML performance in the Vis‐NIR wavelength band. These findings not only deepen our understandings of ML modulation via defect engineering and valence‐state control but also provide a scalable pathway for the design of next‐generation ML materials and intelligent sensing devices.

## 4 Experimental Section

### Synthesis of CaZnOS: Mn^2+^ Powder

A series of CaZnOS: xMn^2+^ (0.0% ≤ x ≤ 9.0%) samples were synthesized using a conventional high‐temperature solid‐state reaction method. High‐purity CaCO_3_ (Aladdin, 99.99%), ZnS (Aladdin, 99.99%), MnCO_3_ (Aladdin, 99.99%), MnO_2_ (Aladdin, 99.99%), and RE_2_O_3_ (Aladdin, 99.99%) were precisely weighed according to stoichiometric ratios. The raw materials were first thoroughly ground in an agate mortar with anhydrous ethanol to ensure uniform mixing. After drying, the homogeneous mixture was transferred into a crucible and subjected to calcination in a tube furnace under a continuous nitrogen flow. The thermal treatment was conducted at 1050 °C for 6 h. After cooling to room temperature, the resulting powder was finely ground to obtain micron‐sized phosphors with uniform particle size for further characterization.

### Fabrication of ML Composites

The phosphor powder was mixed with an epoxy resin precursor solution at a mass ratio of 1:4. The mixture was then cast into silicone molds and dried at 90 °C for 2.5 h, forming cylindrical samples with a diameter of 25 mm and a thickness of 10 mm. A computer‐controlled pressure system applied precise and stable forces to these cylinders, while a spectrometer recorded the resulting ML signals to ensure reliable data acquisition. Additionally, for triboluminescence testing, the phosphor powder was mixed with polydimethylsiloxane (PDMS, Sylgard 184 silicon elastomer, Dow Corning) at a mass ratio of 1:10, cast into molds, and cured at 80 °C for 2 h to fabricate circular films with a thickness of 3 mm.

### Characterization Method

The structural and optical properties of the samples were systematically analyzed using multiscale characterization techniques. XRD analysis was conducted at room temperature using a Rigaku D/max‐III.A diffractometer equipped with a Cu‐Kα_1_ radiation source (*λ* = 1.54 059 Å). Rietveld refinement was performed using the FullProf software to obtain detailed structural parameters. PL and PLE spectra, fluorescence lifetime decay curves, and time‐resolved emission spectra were recorded using an Edinburgh FLS920 fluorescence spectrometer with a 450 W Xe lamp. DR spectra were acquired using an Agilent Cary 60 UV–Vis spectrometer. XPS was conducted using a Thermo Scientific Nexsa spectrometer with an Al Kα monochromatic source to determine the elemental chemical states. Mn K‐edge XAFS measurements were performed at the BL14W beamline (SSRF) using Si (111) monochromators. The spectra were collected in transmission mode using a Bruker 5040 four‐channel SDD detector, while reference samples were measured under the same conditions. Data processing was performed using Athena software. TL measurements were conducted using an FJ427 A1 dosimeter over the temperature range of 327–573 K to investigate defect concentrations. EPR spectroscopy was performed using a Bruker EMXPLUS X‐band spectrometer to characterize oxygen vacancies and Mn valence states, with measurement parameters set at a microwave frequency of 9.84 GHz, modulation amplitude of 5 G, and microwave power of 8 mW. The morphology and elemental distribution of the samples were examined using SEM (JSM2010) and TEM (FEI Tecnai G2 F20). Quantitative elemental analysis was performed using an Agilent 5800 ICP‐OES system operating at 1.0 kW. PFM measurements were carried out on an atomic force microscope (Multimode 8) with an Ag‐coated probe to reveal the local piezoelectric behavior of the samples. For real‐time ML monitoring, a custom‐designed experimental platform was constructed, integrating a CMT1104 pressure testing machine, a QE650pro photon counting spectrometer, and a computer‐based data acquisition system to ensure precise and reproducible ML measurements.

### Calculation Method

All these works were simulated through first‐principles calculations using VASP. The exchange‐correlation energy was treated within the framework of the PBE functional under the GGA. A plane‐wave cutoff energy of 400 eV was used throughout. For Brillouin zone sampling, a Gamma‐centered 2×2×1 Monkhorst–Pack k‐point mesh was adopted. Structural relaxations were performed until the forces on all atoms were less than 0.03 eV Å^−1^, and the electronic self‐consistent field (SCF) calculations were converged to a total energy tolerance of 10^−5^ eV per atom. To evaluate the thermodynamic stability of intrinsic and extrinsic point defects, the defect formation energy, *E^f^* [*X^q^*] was computed using the standard expression

(7) $$E^{f}\;\left[X^{q}\right]=E_{\mathrm{tot}}\;\left[X^{q}\right]-E_{\mathrm{tot}}\left[\mathrm{bulk}\right]-\sum n_{i}\mu_{i}+qE_{F}+E_{\mathrm{corr}}$$

here*, E*_tot_[*X^q^*] and *E^f^* [*X^q^*] denote the total energies of the defective and pristine supercells, respectively. The term *n_i_* represents the number of atoms of species i added to (positive) or removed from (negative) the supercell to form the defect, and µ_*i*_ corresponds to the chemical potential of species i. The Fermi level *E*_F_ referenced to the VBM of the host, represents the electron chemical potential. The correction term *E*_corr_ accounts for finite‐size effects, such as image‐charge interactions and band‐filling effects, particularly important for charged defects in periodic supercells. Charge states of defects are denoted by superscripts (e.g., *q* = 0 for neutral, *q* = +1 for one electron removed, and *q* = −1 for one electron added). While only neutral defects exist in metals, multiple charge states are possible in semiconductors or insulators through electron exchange with reservoirs.

The chemical potentials µ_*i*_ are subject to thermodynamic stability conditions to prevent the precipitation of competing phases. Under S‐rich and Ca‐rich synthesis conditions, the chemical potentials satisfy the following constraints

(8) $$\mu_{\mathrm{Ca}}+\mu_{\mathrm{Zn}}+\mu_{O}+\mu_{S}=E{(}_{\mathrm{CaZnOS}})$$

(9) $$\mu_{\mathrm{Ca}}+\mu_{O}\leq E{(}_{\mathrm{CaO}})$$

(10) $$\mu_{\mathrm{Zn}}+\mu_{S}\leq E{(}_{\mathrm{ZnS}})$$

(11) $$\mu_{\mathrm{Zn}}\leq E{(}_{\mathrm{Zn}})$$

Given that synthesis was conducted in a N_2_ atmosphere (oxygen‐deficient), the oxygen chemical potential µ_O_ is not derived from one‐half the O_2_ molecular energy but instead reflects O‐poor growth conditions. The sulfur chemical potential µ_S_ can be approximated using the standard‐state energy of elemental sulfur. These constraints enable accurate estimation of the defect formation energies and their likelihood under experimental synthesis conditions.

### ANN Training

To establish a reliable mapping between mechanical stimulation and ML emission patterns, 900 ML images were collected using the orthodontic sensor under uniform 6 N stress across nine occlusal regions. Each region contributed 100 labeled images, with 720 used for training and 180 for testing. Images were preprocessed by downscaling to 28 × 28 pixels, converting to grayscale, normalizing pixel intensities to [0,1], and flattening into 784‐dimensional vectors. A neural network was implemented in TensorFlow 2.10 and trained on an NVIDIA RTX 3090 GPU. The architecture included an input layer, three fully connected hidden layers with ReLU activation, and a 9‐neuron Softmax output layer. Training used categorical cross‐entropy loss and the Adam optimizer (learning rate 0.001, *β*_1_  =  0.9, *β*_2_  =  0.999), with a batch size of 32 over up to 50 epochs. Dropout (rates: 0.3, 0.2, 0.2) and early stopping were applied to prevent overfitting. Model performance was evaluated using a held‐out test set to verify accuracy and generalization.

## Conflict of Interest

The authors declare no conflict of interest.

## Supporting information



Supporting Information

Supplemental Movie 1

Supplemental Movie 2

## Data Availability

The data that support the findings of this study are available from the corresponding author upon reasonable request.
