# Supplementary material for: Boosting Mechanoluminescence Performance in Doped CaZnOS by the Facile Self‐Reduction Approach
Source: Adv Mater. 2025 Sep 26;38(3):e11643. doi: 10.1002/adma.202511643 (PMC12801364; doi:10.1002/adma.202511643)
Supplement: Supplementary file 1 — Supporting Information [file ADMA-38-e11643-s003.pdf]

ADVANCED MATERIALS

Supporting Information

for *Adv. Mater.*, DOI 10.1002/adma.202511643

Boosting Mechanoluminescence Performance in Doped CaZnOS by the Facile Self-Reduction Approach

Shengbin Xu, Yao Xiao, Puxian Xiong, Pan Zheng, Sheng Wu, Xuesong Wang, Yumin Yin, Haiqiang Fang, Chengan Wang, Yuexi Lu, Enhai Song and Jiulin Gan**

Supporting Information

Boosting Mechanoluminescence Performance in doped CaZnOS by the Facile Self-reduction Approach

Shengbin Xu[1,#], Yao Xiao[1,#], Puxian Xiong[2,#,], Pan Zheng[1], Sheng Wu[3], Xuesong Wang[1], Yumin Yin[1], Haiqiang Fang[1], Chengan Wang[1], Yuexi Lu[1], Enhai Song[1] and Jiulin Gan[1,*]*

1 State Key Laboratory of Luminescent Materials and Devices; Institute of Optical Communication Materials; Guangdong Engineering Technology Research and Development Center of Special Optical Fiber Materials and Devices; Guangdong Provincial Key Laboratory of Fiber Laser Materials and Applied Techniques; South China University of Technology, Guangzhou 510640, China.

2 Department of Electrical and Electronic Engineering, The University of Hong Kong, Hong Kong, 999077, China.

3 Guangdong Basic Research Center of Excellence for Structure and Fundamental Interactions of Matter; Guangdong Provincial Key Laboratory of Quantum Engineering and Quantum Materials; Guangdong-Hong Kong Joint Laboratory of Quantum Matter; Frontier Research Institute for Physics; School of Physics, South China Normal University; Guangzhou 510006, China.

# Equally contributed to this work.

* Corresponding author.

E-mail:

Dr. P.X. Xiong: pxxiong@hku.hk;

Prof. J.L. Gan: msgan@scut.edu.cn;

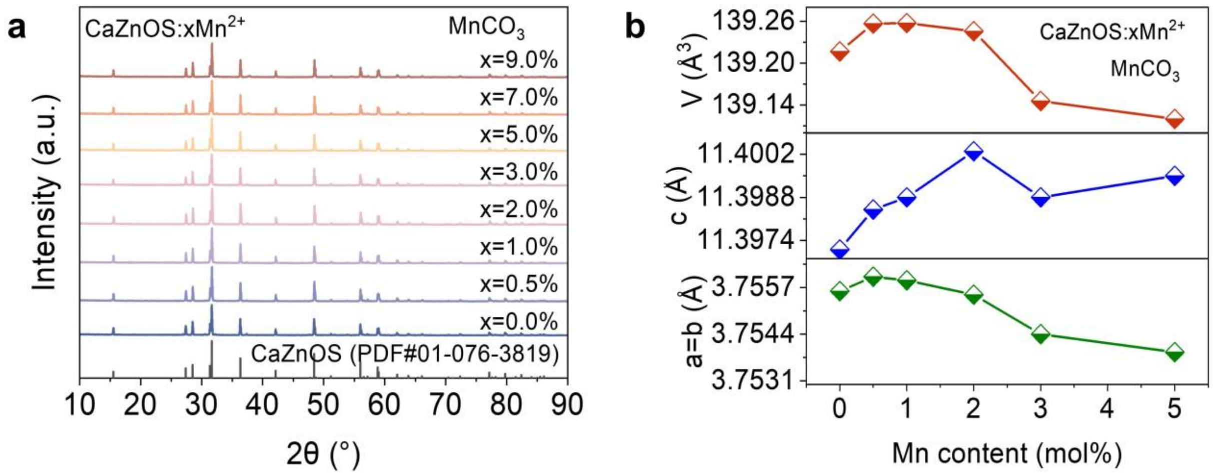

Figure S1. (a) XRD patterns of CaZnOS: xMn^{2+} (x = 0, 0.5%, 1.0%, 2.0%, 3.0%, 5.0%, 7.0%, 9.0%) synthesized from $MnCO_3$ and the standard PDF card of CaZnOS. (b) Lattice parameters (a, b, c) and unit cell volume (V) at varying Mn^{2+} doping concentrations.

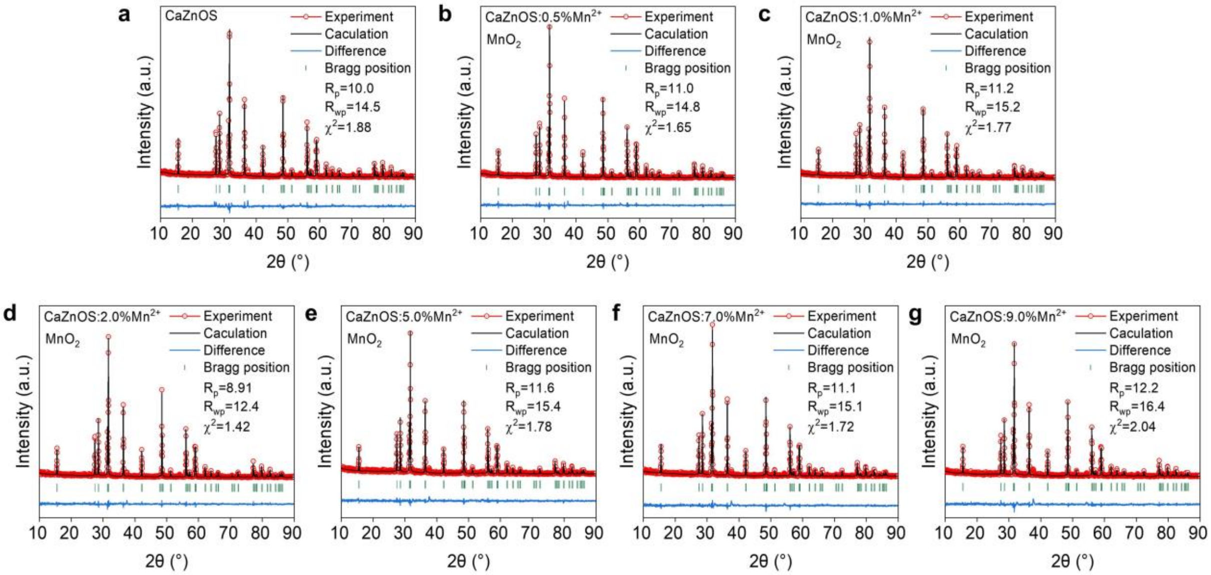

Figure S2. Rietveld refinement patterns of CaZnOS: xMn^{2+} (x=0-9.0%) synthesized from MnO_2. The refinement parameters have been marked on the diagram. (a) x=0; (b) x=0.5%; (c) x=1.0%; (d) x=2.0%; (e) x=5.0%; (f) x=7.0%; (g) x=9.0%.

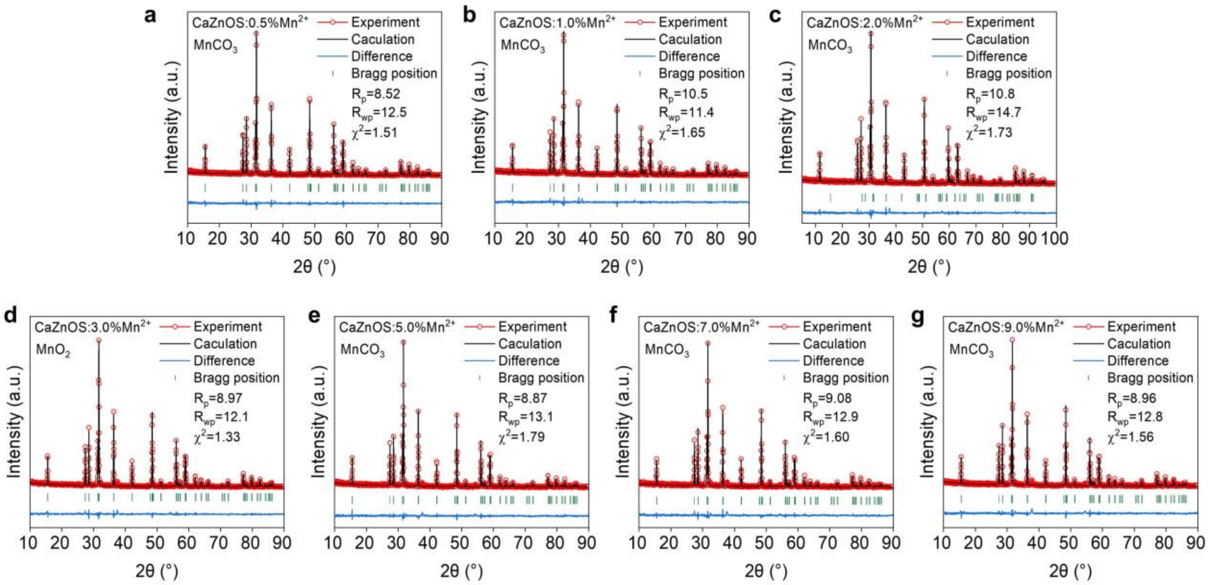

Figure S3. Rietveld refinement patterns of CaZnOS: xMn^{2+} (x=0.5-9.0%) synthesized from $MnCO_3$. The refinement parameters have been marked on the diagram. (a) x=0.5%; (b) x=1.0%; (c) x=2.0%; (d) x=3.0%; (e) x=5.0%; (f) x=7.0%; (g) x=9.0%.

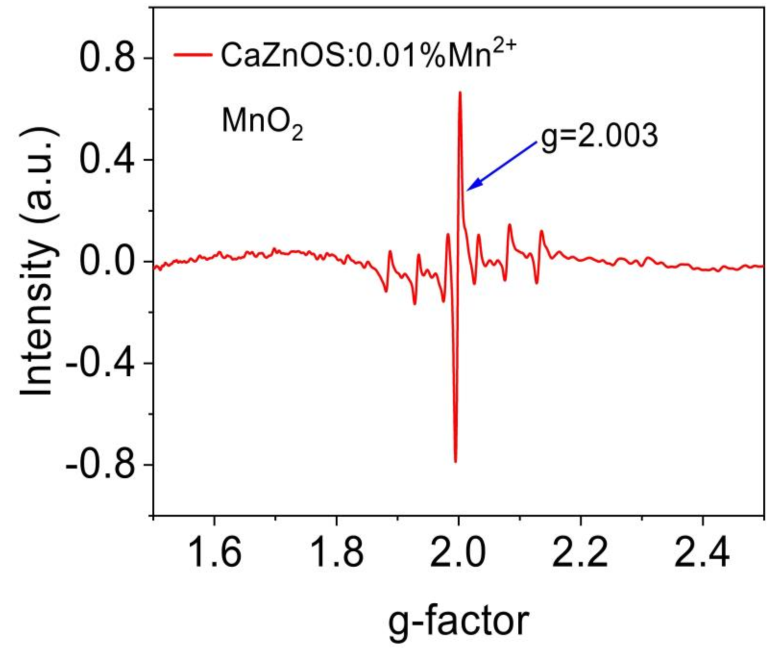

Figure S4. EPR spectra of CaZnOS:0.01%Mn^{2+} regarding g-factor synthesized from MnO_2. In addition to the characteristic six-level hyperfine curve of Mn^{2+}, the strong signal peak corresponding to the oxygen vacancy with g = 2.003 is also detected.

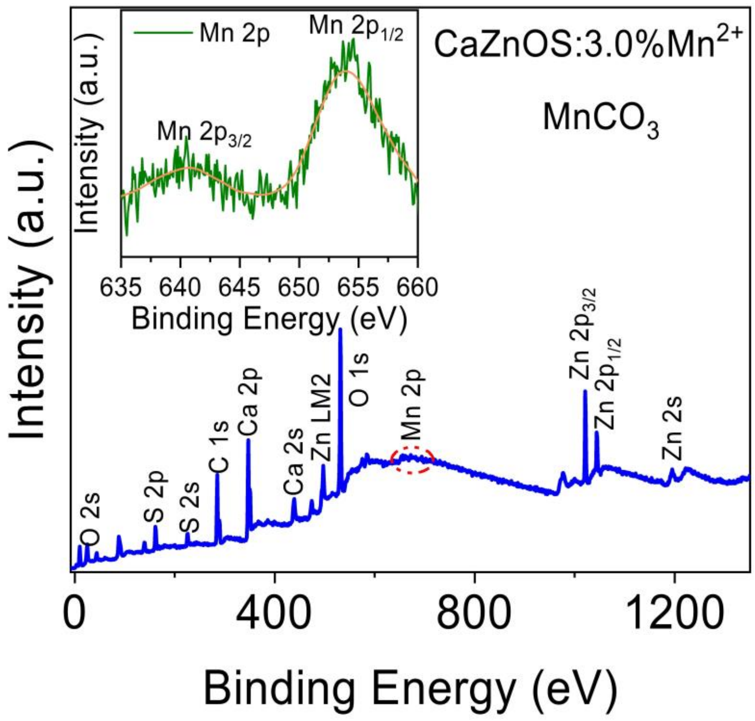

Figure S5. The full XPS spectrum of CaZnOS: 3.0%Mn^{2+} using $MnCO_3$ as Mn source and the insert shows the high-resolution 2p orbital image of Mn.

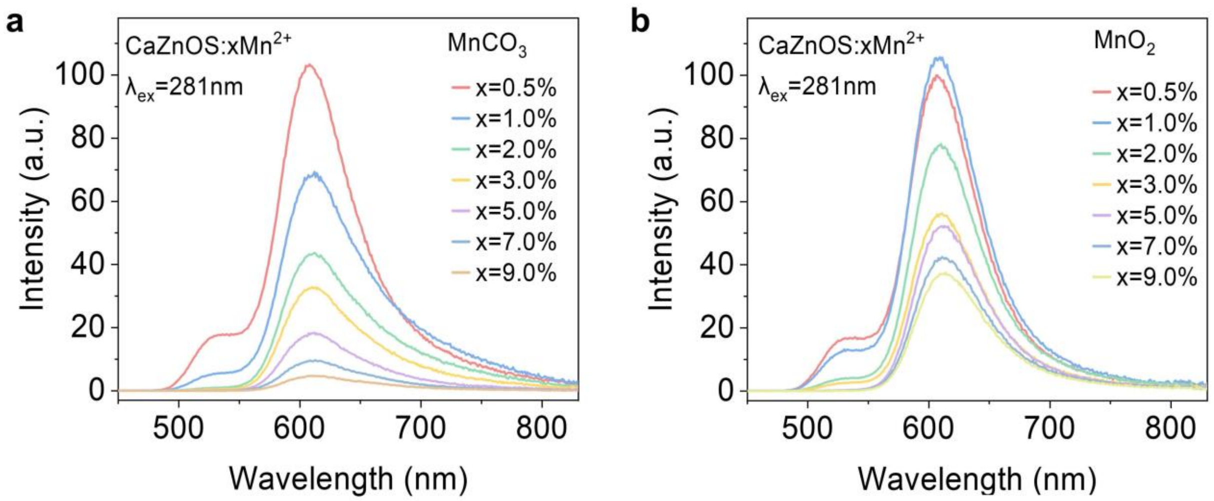

Figure S6. (a) PL (λ_{ex}=281 nm) spectra of CaZnOS: xMn^{2+} (x=0, 0.5%, 1.0%, 2.0%, 3.0%, 5.0%, 7.0%, 9.0%) using $MnCO_3$ as the Mn source. (b) PL (λ_{ex}=281 nm) spectra of CaZnOS: xMn^{2+} (x=0, 0.5%, 1.0%, 2.0%, 5.0%, 7.0%, 9.0%) using MnO_2.

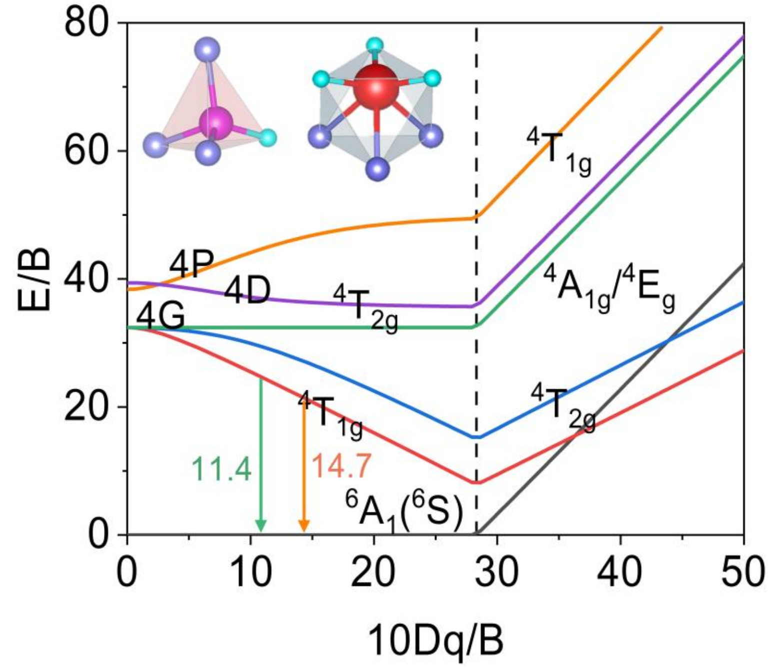

Figure S7. Tanabe-Sugano energy-level diagram of Mn^{2+} ion (d^5 electronic configuration). The calculation results indicate that the green light emission at 534 nm originates from Mn2+ occupying the 4-coordinated Zn site, and the crystal field strength at this site is 10Dq/B = 11.4. The orange light emission at 608nm originates from Mn2+ occupying the 6-coordinated Ca site, and the crystal field strength at this site is 10Dq/B = 14.7.

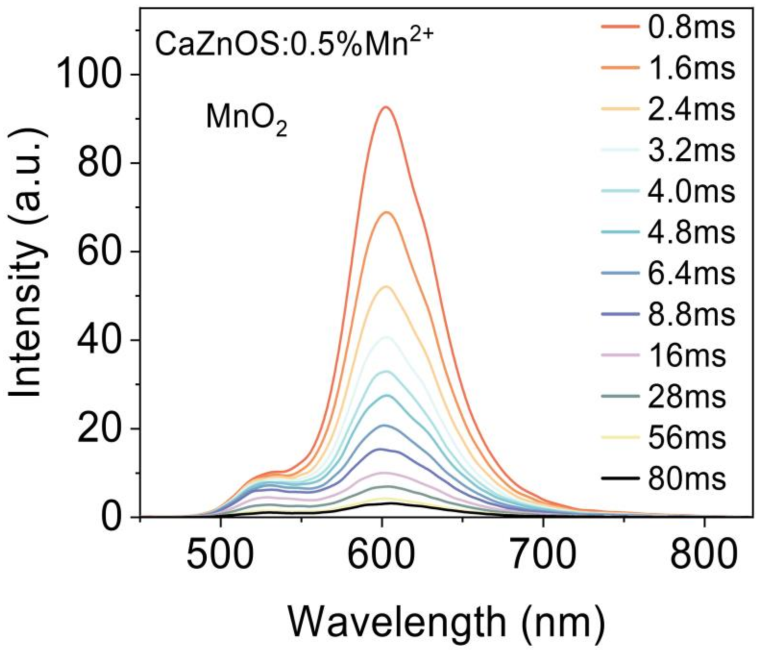

Figure S8. Time-resolved PL (λ_{ex}=281 nm) spectra of CaZnOS: 0.5%Mn^{2+} synthesized using MnO_2 from 0.8 to 80 ms.

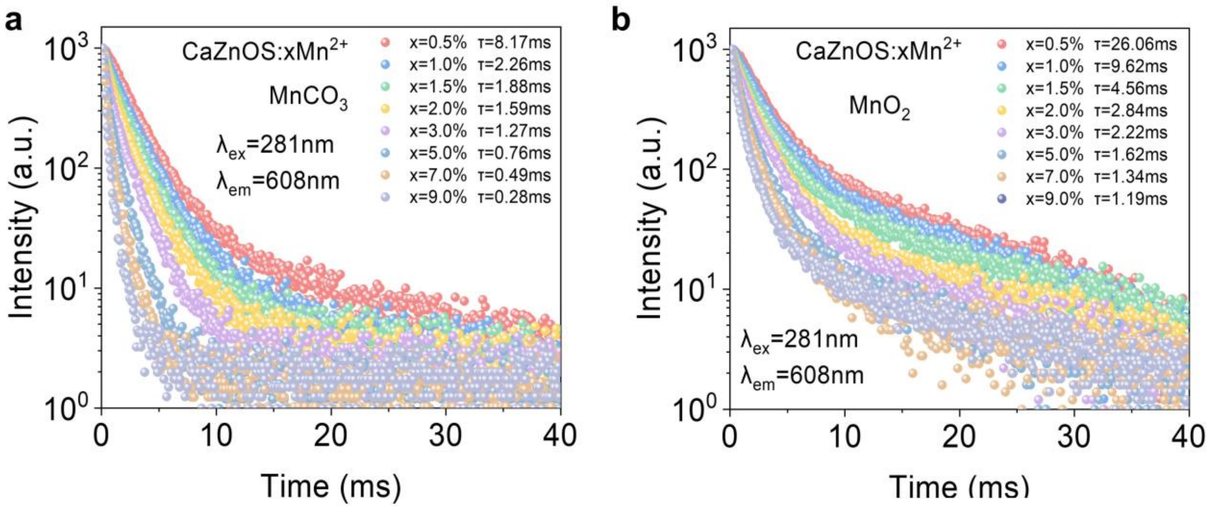

Figure S9. (a) Fluorescence lifetime curves of CaZnOS: xMn^{2+} (x=0, 0.5%, 1.0%, 2.0%, 3.0%, 5.0%, 7.0%, 9.0%) using $MnCO_3$ as the Mn sources (λ_{ex}=281 nm, λ_{em}=608 nm). (b Fluorescence lifetime curves of CaZnOS: xMn^{2+} (x=0, 0.5%, 1.0%, 2.0%, 3.0%, 5.0%, 7.0%, 9.0%) using MnO_2 (λ_{ex}=281 nm, λ_{em}=608 nm).

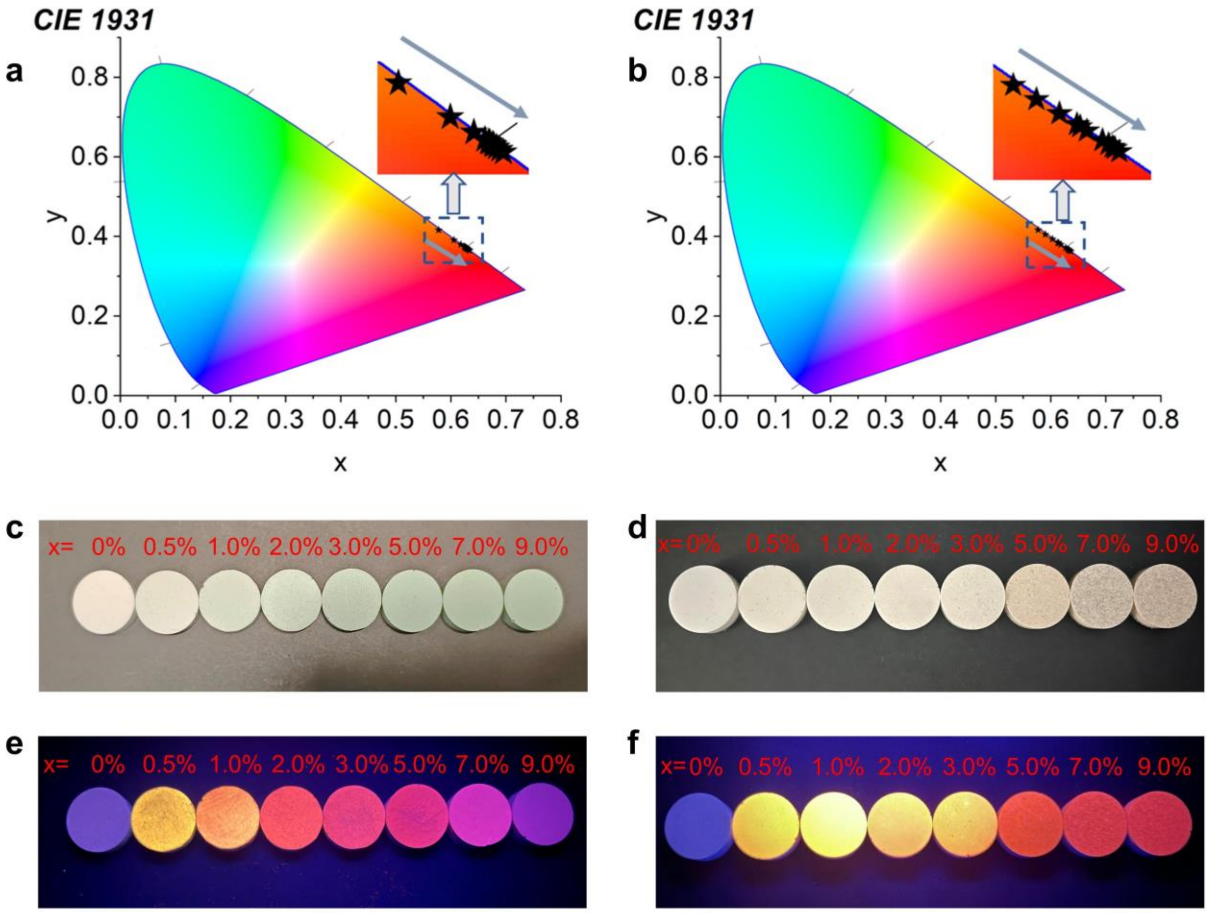

Figure S10. (a, b) The CIE coordinates shift with the variation of Mn content using $MnCO_3$ and MnO_2 as the Mn sources respectively. (c, d) Images of the two batches of samples under natural light. (e, f) The PL colors of the two batches of samples under a 254 nm ultraviolet light.

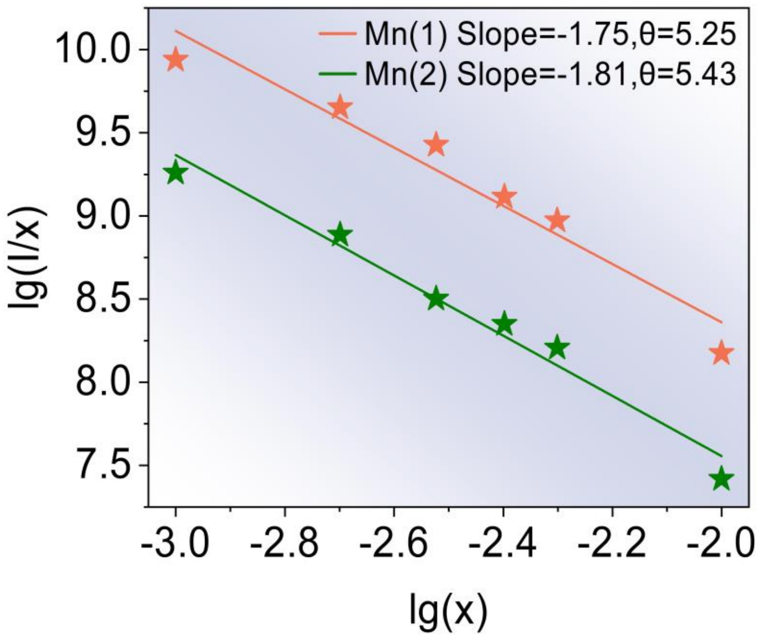

Figure S11. Fitted curve (log (I/x) vs log (x)) of dual PL peaks of CaZnOS: xMn^{2+} (x=0.5%, 1.0%, 2.0%, 3.0%,5.0%, 7.0%). Mn (1) and Mn (2) respectively represent the two emission peaks located at 534 nm and 608 nm. The θ value close to 6 indicates that the dipole-dipole interaction is the cause of the quenching of these two emission peaks.

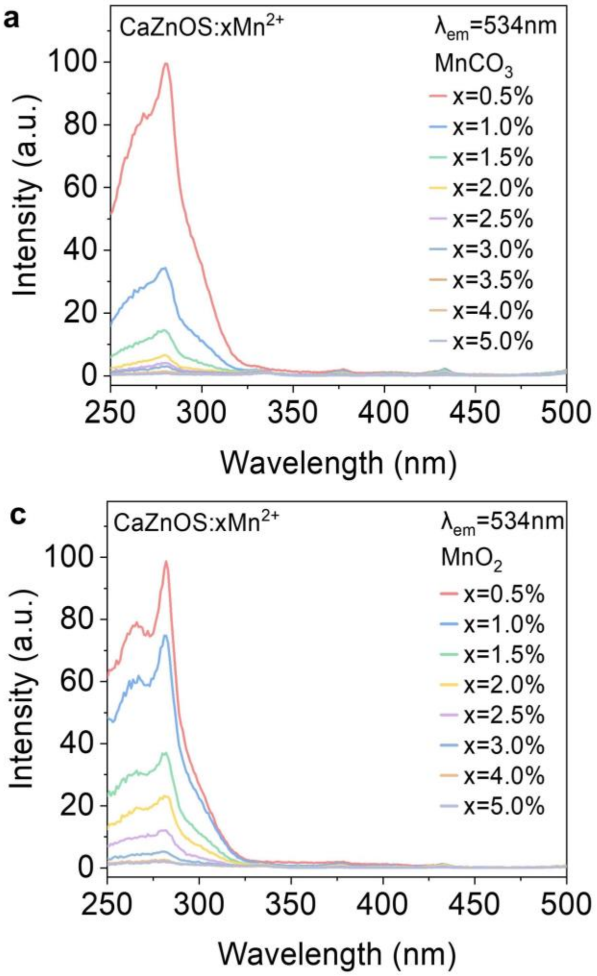

Figure S12. (a, b) PLE spectra of CaZnOS: xMn^{2+} (x=0, 0.5%, 1.0%, 2.0%, 3.0%, 5.0%, 7.0%, 9.0%) monitored at 534 nm and 608 nm synthesized from $MnCO_3$. (c, d) PLE spectra of CaZnOS: xMn^{2+} (x=0, 0.5%, 1.0%, 2.0%, 3.0%, 5.0%, 7.0%, 9.0%) monitored at 534 nm and 608 nm synthesized from MnO_2.

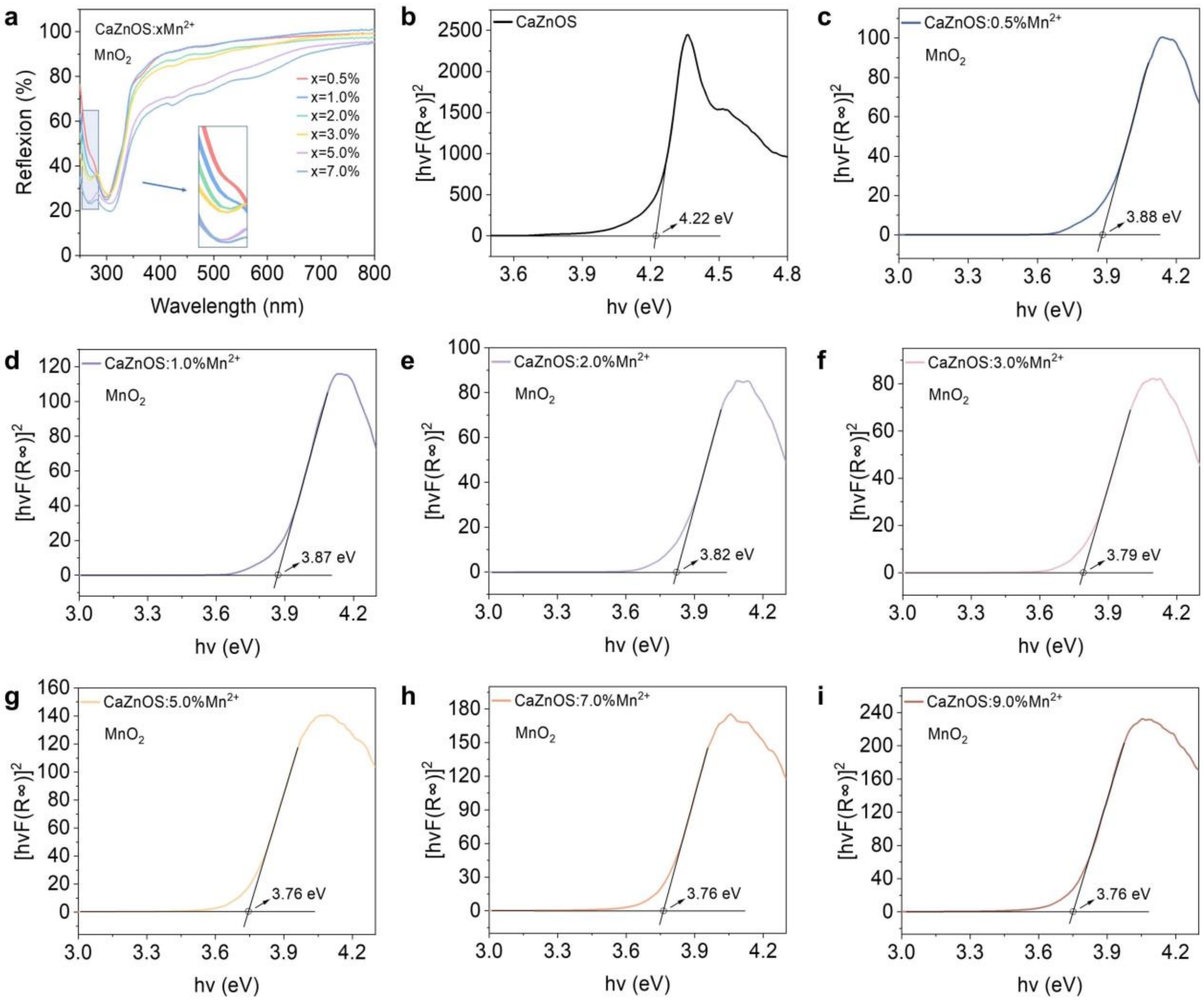

Figure S13. (a) Diffuse reflectance spectra of CaZnOS: xMn^{2+} (x=0, 0.5%, 1.0%, 2.0%, 3.0%, 5.0%, 7.0%) synthesized from MnO_2. (b-i) Optical band gap of CaZnOS: xMn^{2+} (x=0, 0.5%, 1.0%, 2.0%, 3.0%, 5.0%, 7.0%, 9.0%) calculated by Kubelka–Munk function.

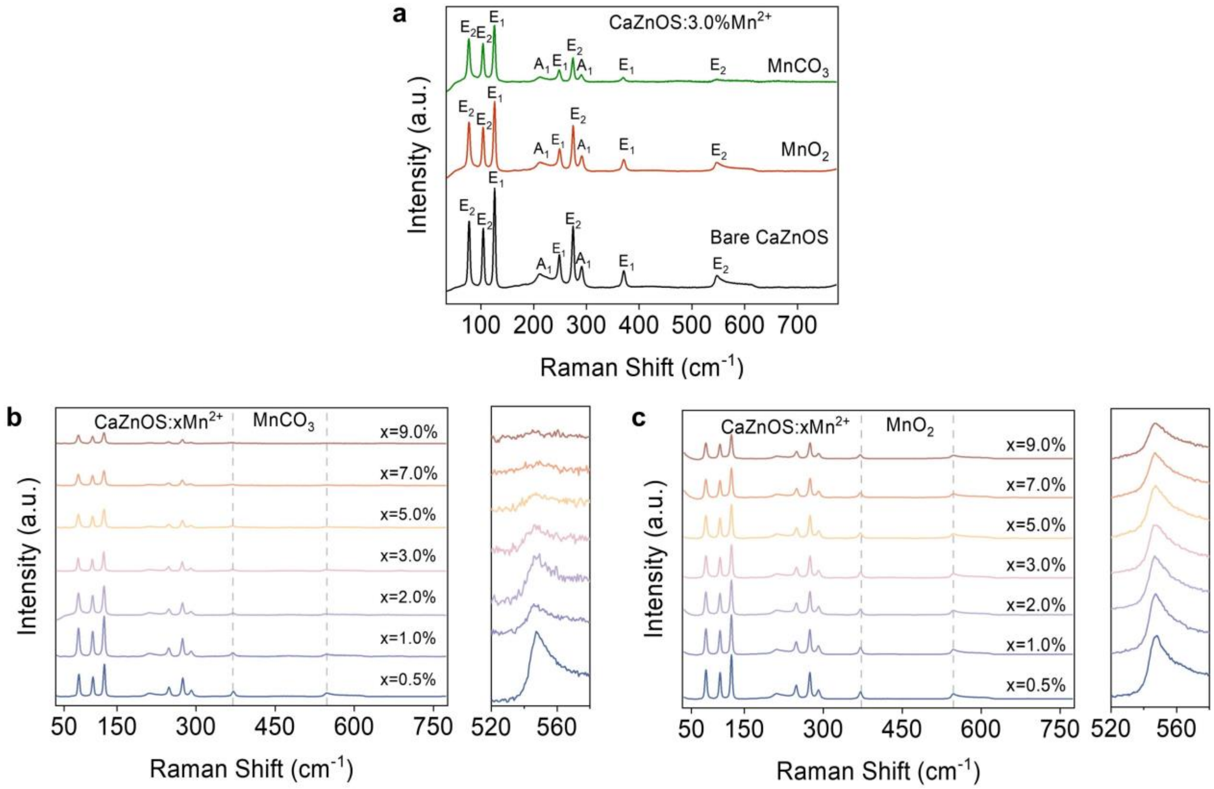

Figure S14. (a) Comparison of Raman spectra of CaZnOS: 3.0%Mn^{2+} and pristine CaZnOS. (b) Raman spectra and the intensity of the vibration peaks of CaZnOS: xMn^{2+} (x=0.5%, 1.0%, 2.0%, 3.0%, 5.0%, 7.0%, 9.0%) synthesized from $MnCO_3$. (c) Raman spectra and the intensity of the vibration peaks of CaZnOS: xMn^{2+} (x=0.5%, 1.0%, 2.0%, 3.0%, 5.0%, 7.0%, 9.0%) synthesized from MnO_2.

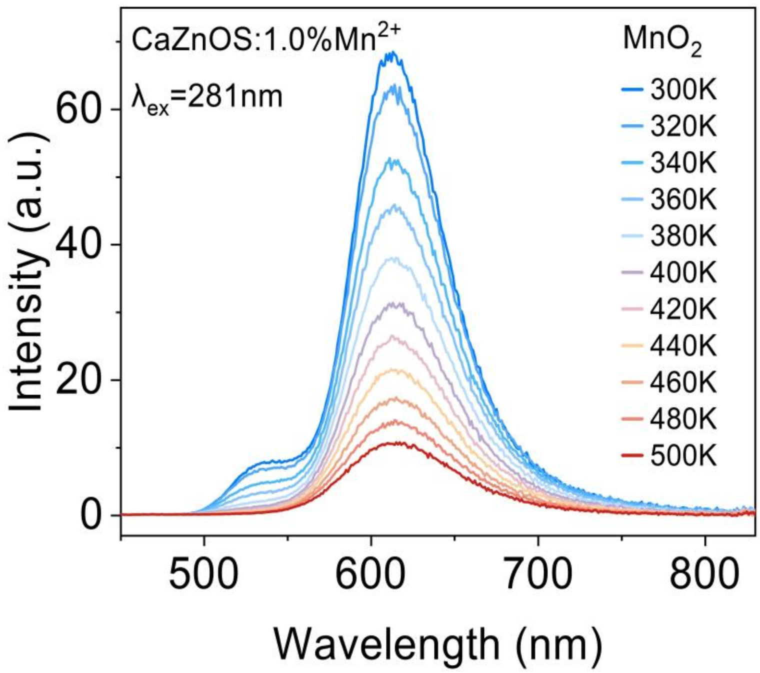

Figure S15. Temperature-dependent PL (λ_{ex}=281 nm) spectra of self-reduced CaZnOS: 1.0%Mn^{2+} from 300-500K.

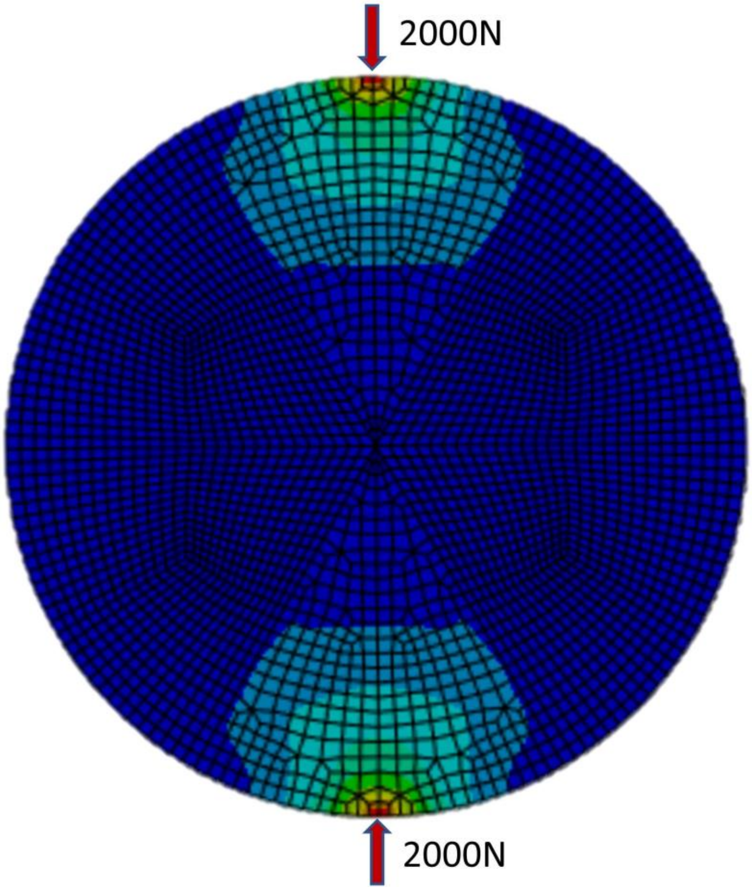

Figure S16. The stress distribution of the epoxy resin cylinder simulated through finite element analysis when subjected to a pressure of 2000N.

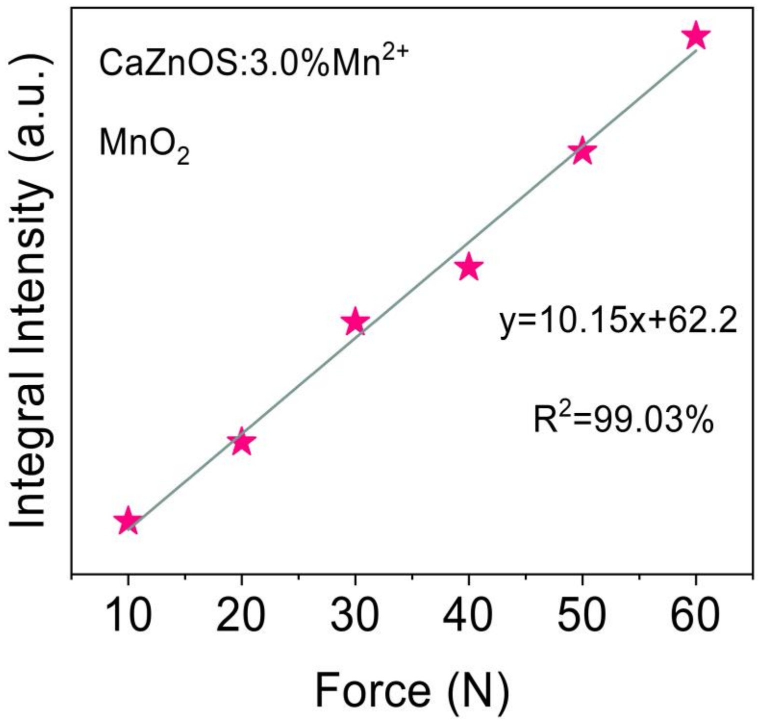

Figure S17. Linear fitting curve of ML integrated intensity of CaZnOS: 3.0%Mn^{2+} under different stress magnitudes (10, 20, 30, 40, 50 and 60 N). The ML intensity is strongly correlated with the magnitude of the applied force (R^2=99.03%).

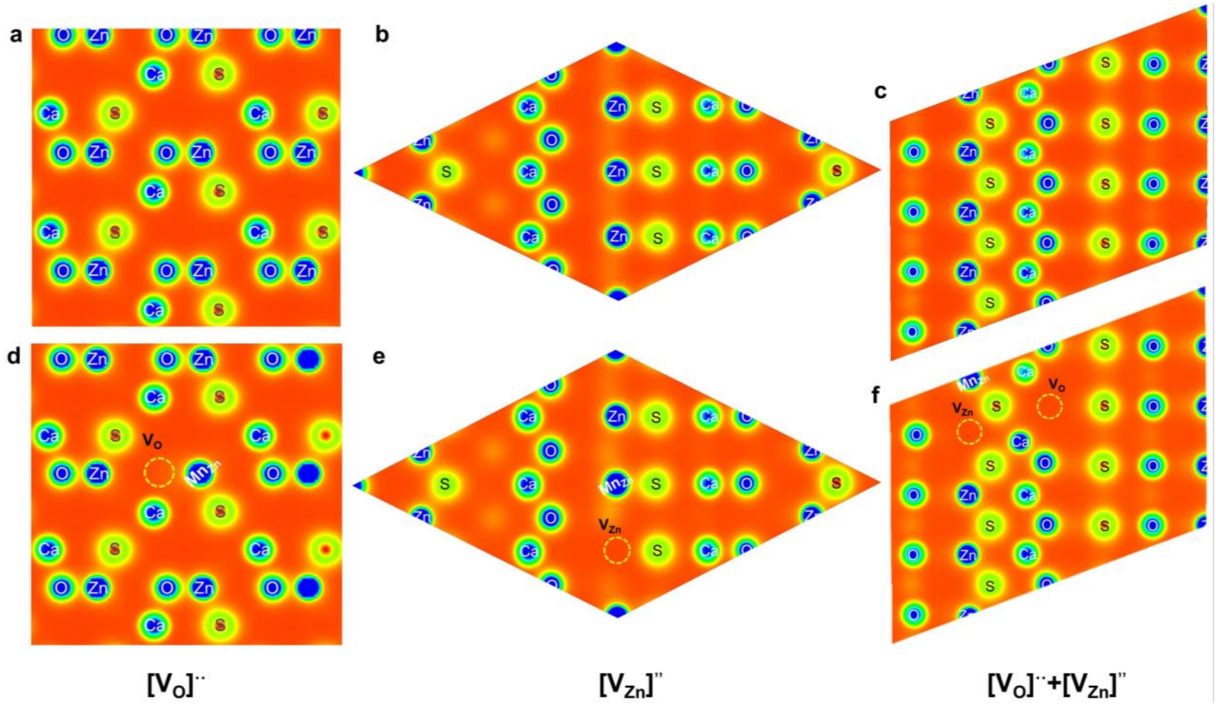

Figure S18. (a–c) Simulated 2D charge density distributions of pristine CaZnOS at various crystallographic positions, illustrating the local electronic environment within the host lattice. (d–f) 2D charge density maps showing the influence of distinct defect types on the electronic structure, corresponding to specific crystallographic planes of CaZnOS. These visualizations reveal the charge redistribution and potential electron trapping behavior induced by defect formation, which contribute to the enhanced piezoelectric and ML responses.

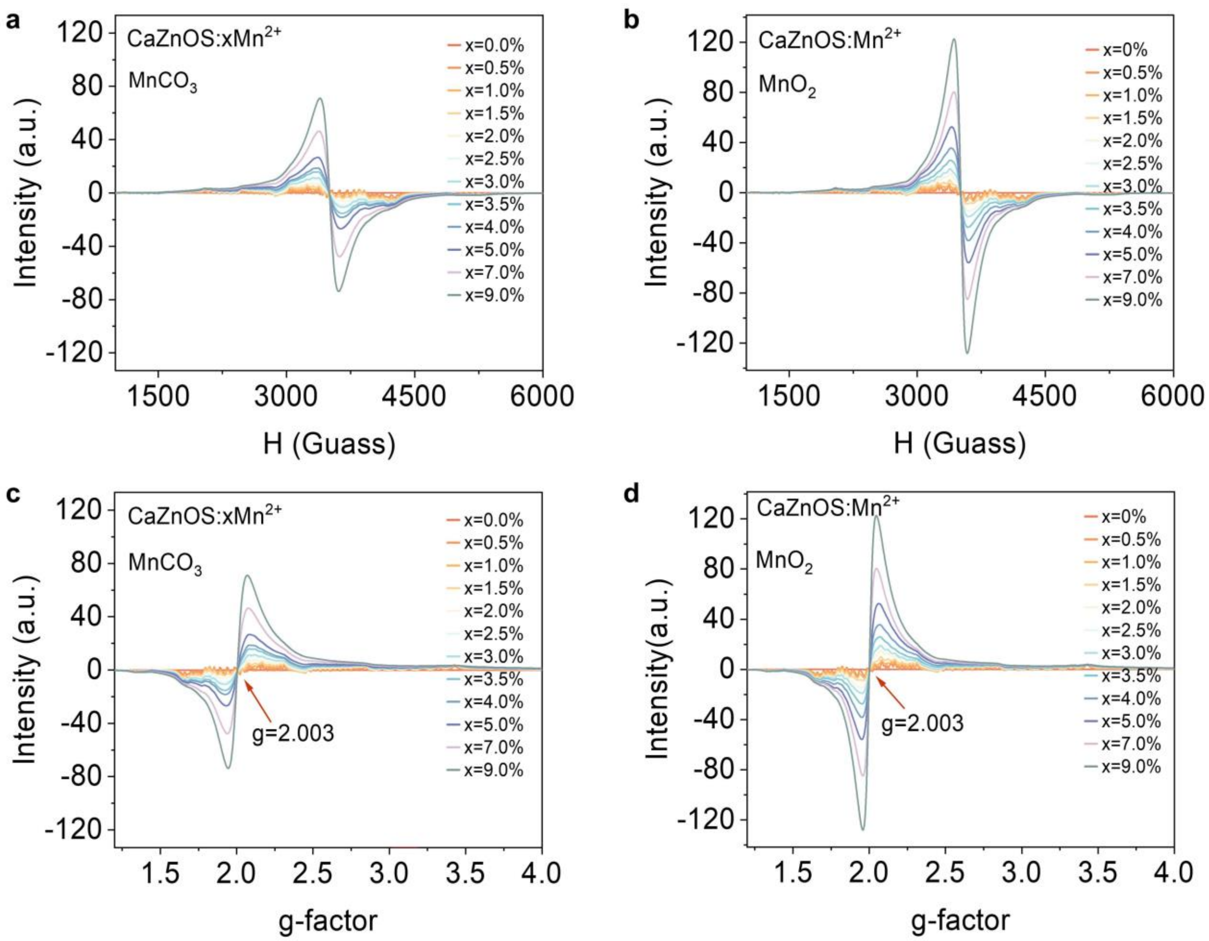

Figure S19. (a-b) EPR (1000-6000 G) spectra of CaZnOS: xMn^{2+} (x=0-9.0%) using $MnCO_3$ and MnO_2 as the Mn sources respectively. (c-d) EPR (g=1.2-4.0) spectra of CaZnOS: xMn^{2+} regarding g-factor using $MnCO_3$ and MnO_2 as the Mn sources respectively.

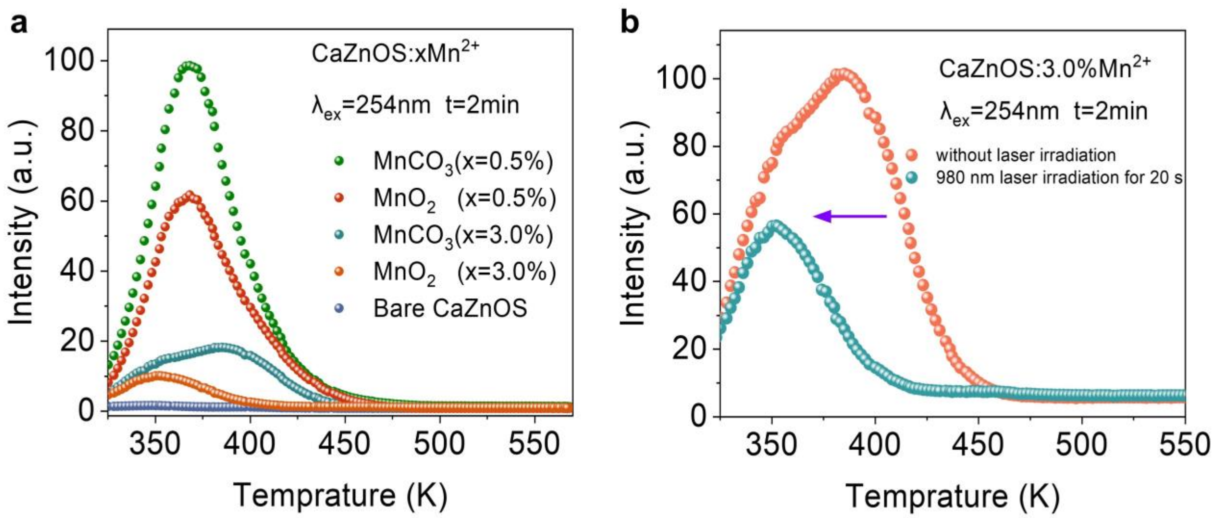

Figure S20. (a) TL curves of CaZnOS: xMn^{2+} (x=0, 0.5%, 3.0%) with and without self-reduction from 300-600K. (b) TL curves of CaZnOS: 3.0%Mn^{2+} with and without 980 nm laser irradiation. All the samples were pre-irradiated with 254 nm ultraviolet light for 2 minutes before the TL test to charge up.

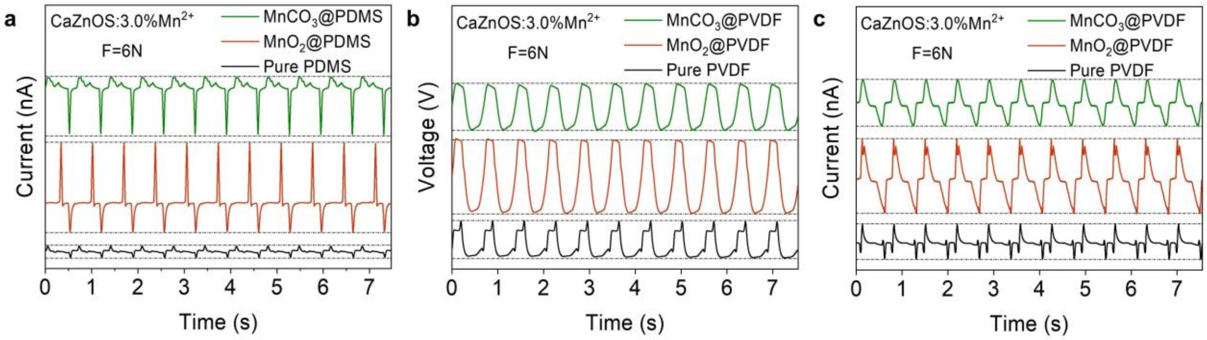

Figure S21. (a) Piezoelectric signals of CaZnOS: 3.0%Mn^{2+} with and without self-reduction embedded in PDMS under 6 N cyclic loading, pure PDMS film was used as the control group. (b-c) Piezoelectric signals of CaZnOS: 3.0%Mn^{2+} with and without self-reduction embedded in PVDF under 6 N cyclic loading, pure PVDF film was used as the control group.

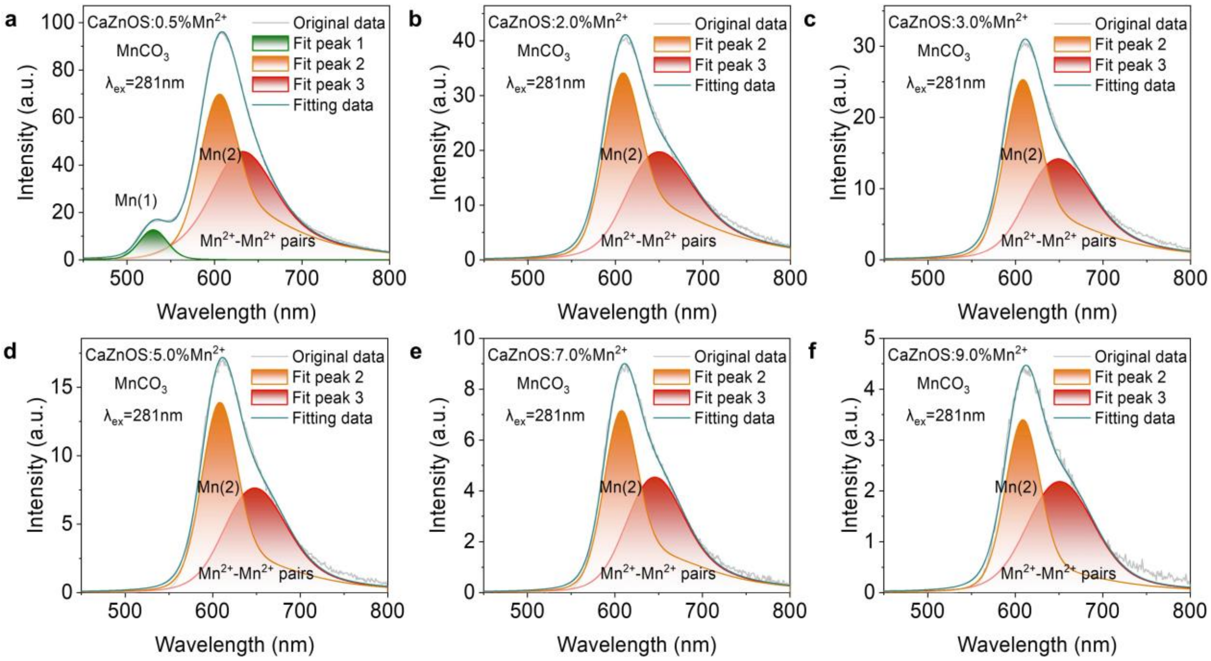

Figure S22. Peak-fitting of the PL spectra under 281 nm excitation of CaZnOS: x%Mn^{2+}. Among the components obtained through deconvolution, the emission peak Mn (1) represents the green light emission at 534 nm, the emission peak Mn (2) represents the orange light emission at 608 nm, and the deep red broad peak generated by Mn^{2+}-Mn^{2+}. (a) x=0.5%; (b) x=2.0%; (c) x=3.0%; (d) x=5.0%; (e) x=7.0%; (f) x=9.0%.

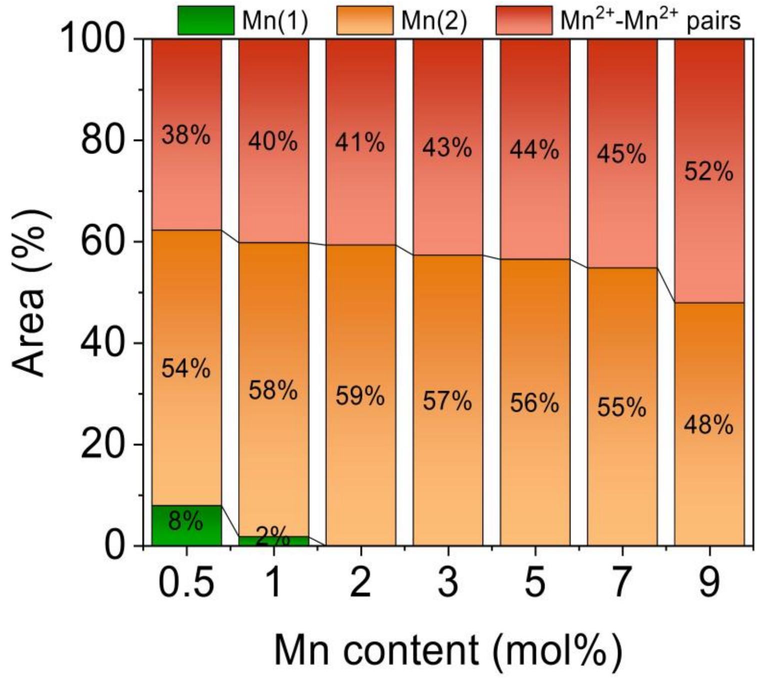

Figure S23. The proportion of the peak areas in the PL (λ_{ex}=281 nm) spectra under different Mn^{2+} doping concentrations.

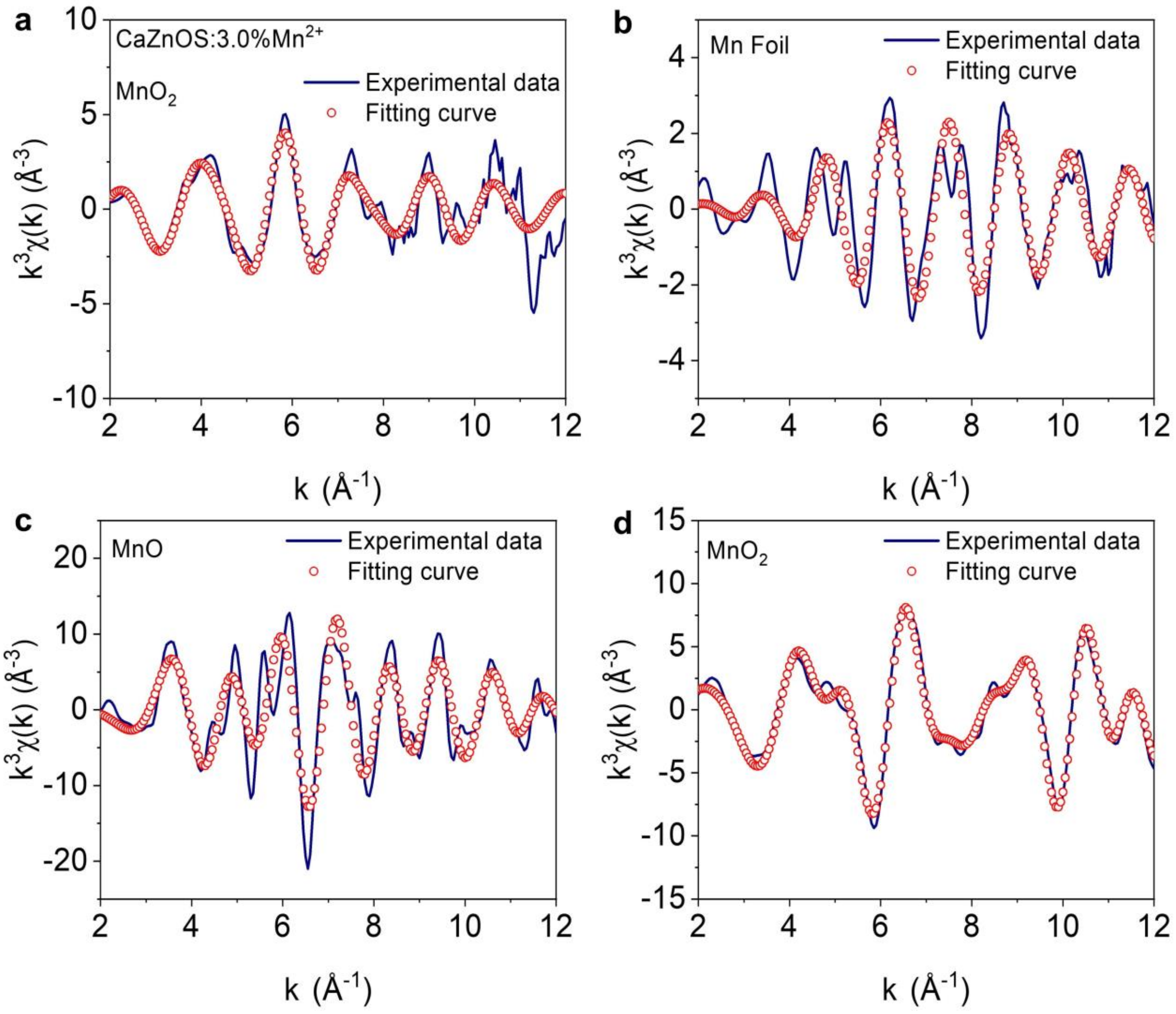

Figure S24. FT-EXAFS spectra of CaZnOS: 3.0%Mn^{2+} with self-reduction, Mn foil, MnO and MnO_2 standard samples.

The XAFS data were processed according to the standard procedures using the Athena module implemented in the IFEFFIT software packages. The EXAFS spectra were obtained by subtracting the post-edge background from the overall absorption and then normalizing with respect to the edge-jump step. Subsequently, the $\chi(k)$ data of were Fourier transformed to real (R) space using a hanning windows (dk = 1.0 $Å^{-1}$) to separate the EXAFS contributions from different coordination shells. To obtain the quantitative structural parameters around central atoms, least-squares curve parameter fitting was performed using the ARTEMIS module of IFEFFIT software packages.

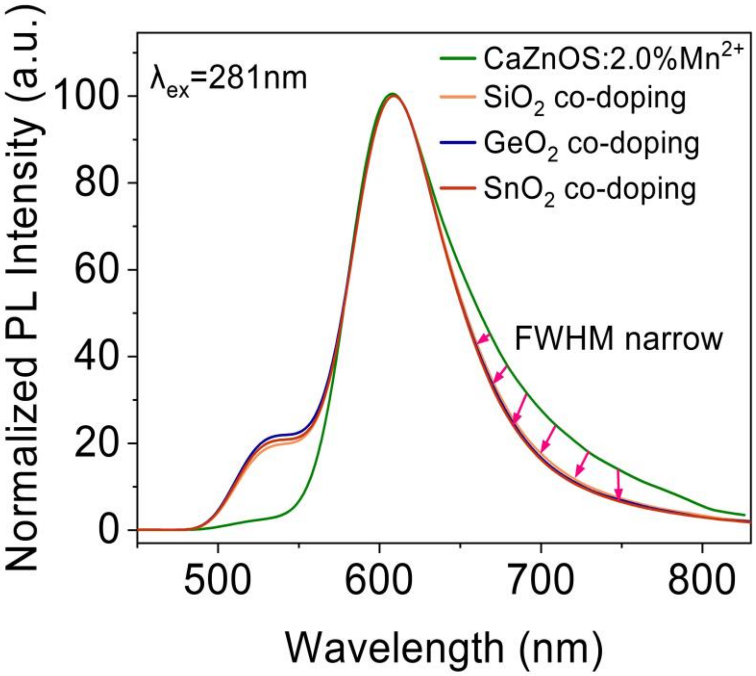

Figure S25. Normalized PL (λ_{ex}=281 nm) curves of CaZnOS: 2.0%Mn^{2+} co-doped with Si^{4+}, Ge^{4+}, Sn^{4+} without self-reduction. Due to the partial inhibition of the formation of Mn^{2+}-Mn^{2+} pairs, the PL spectra show a decrease in FWHM.

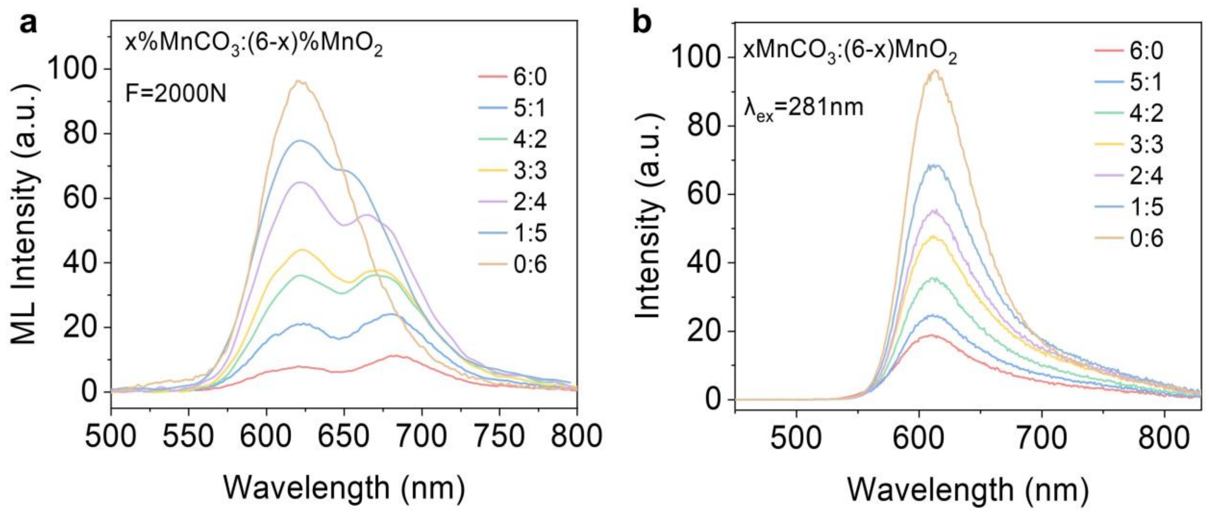

Figure S26. (a) ML spectra of CaZnOS: x%$MnCO_3$/(6-x)%MnO_2 (x=0, 1, 2, 3, 4, 5, 6) under 281 nm excitation. (b) PL spectra of CaZnOS: x$MnCO_3$/(6-x) MnO_2 (x=0, 1, 2, 3, 4, 5, 6) under 2000 N compression.

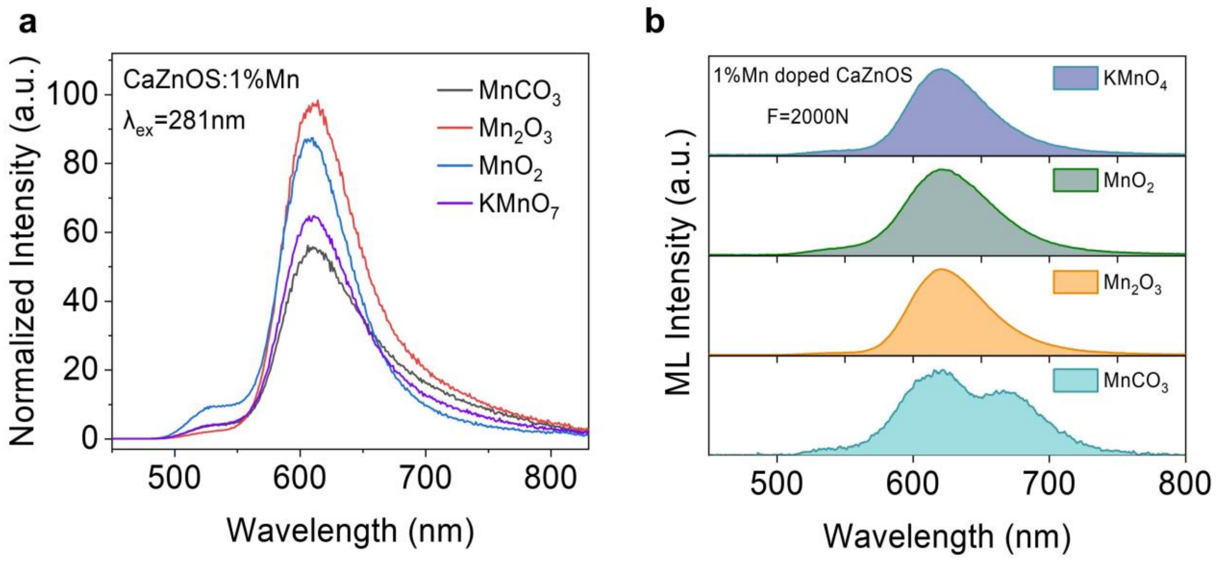

Figure S27. (a) PL spectra of CaZnOS: 1.0%Mn^{2+} using $MnCO_3$, Mn_2O_3, MnO_2, $KMnO_4$ respectively under 281 nm excitation. (b) ML spectra of CaZnOS: 1.0%Mn^{2+} using $MnCO_3$, Mn_2O_3, MnO_2, $KMnO_4$ respectively under 2000 N compression.

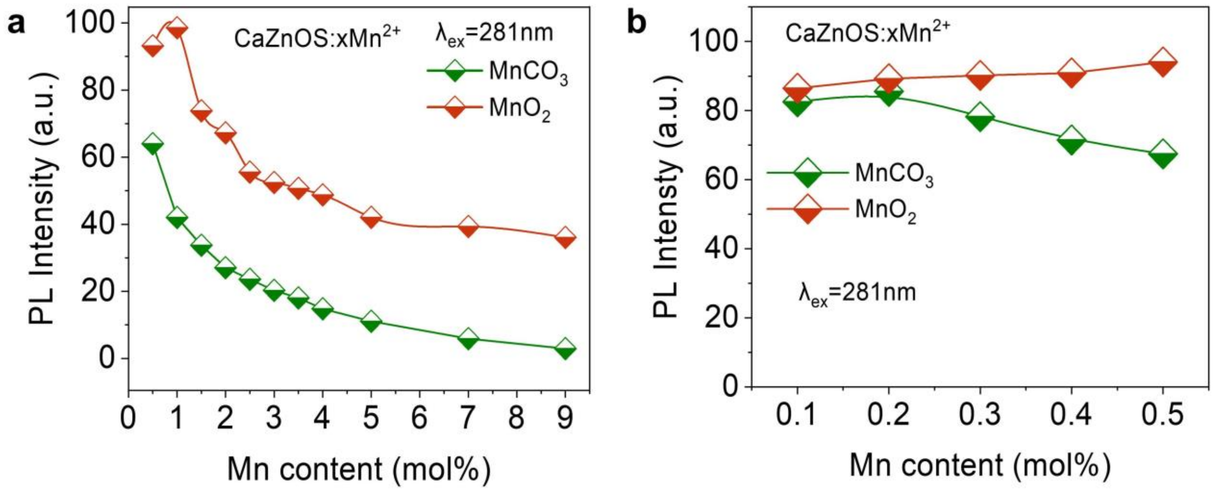

Figure S28. Comparison of the intensity of the PL peak of CaZnOS: xMn^{2+} with and without self-reduction under 281 nm excitation. (a) x=0.5-9.0%; (b) x=0.1-0.5%

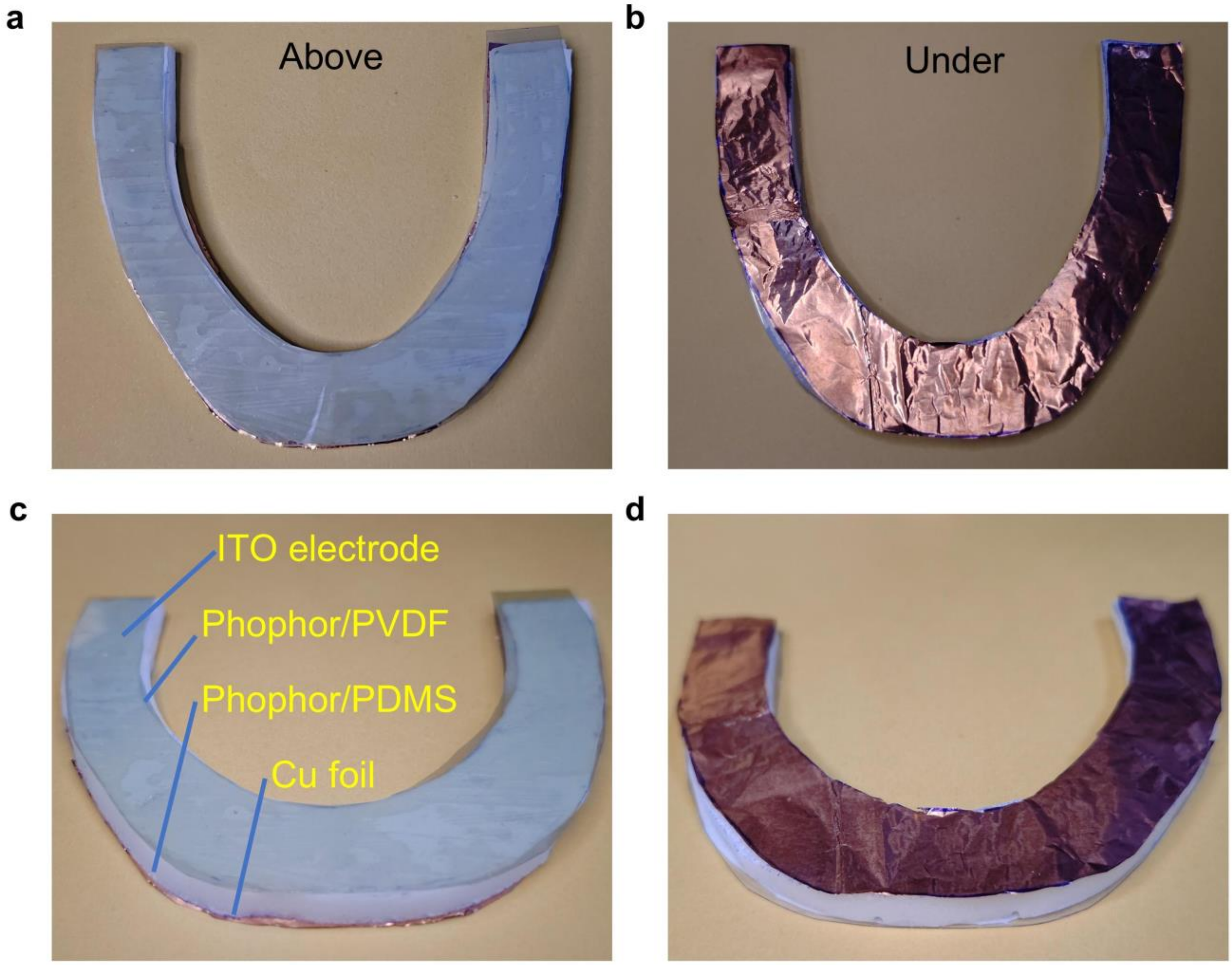

Figure S29. The physical images of the multi-layer orthodontic sensor from different perspectives.

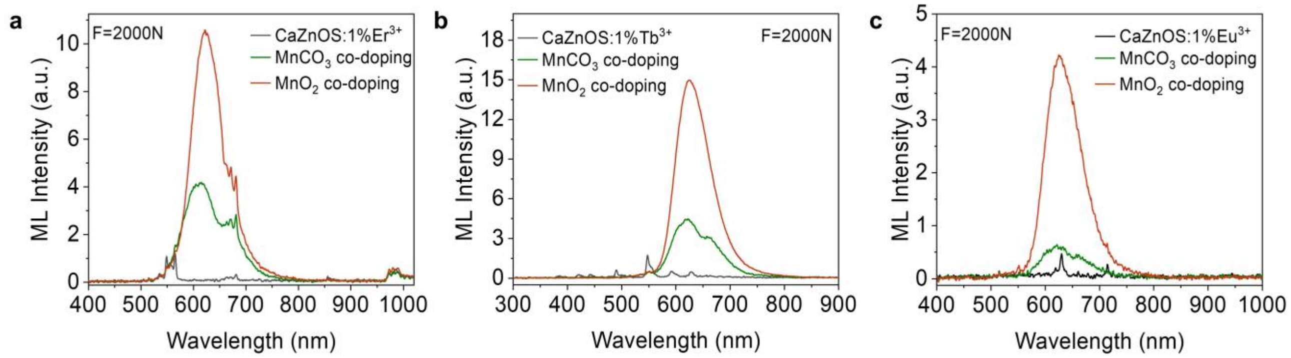

Figure S30. ML spectra of RE irons (Er^{3+}, Tb^{3+}, Eu^{3+}) co-doped with Mn^{4+} in CaZnOS. And the single-doped RE ions system and the $MnCO_3$/RE co-doping system were set as control groups.

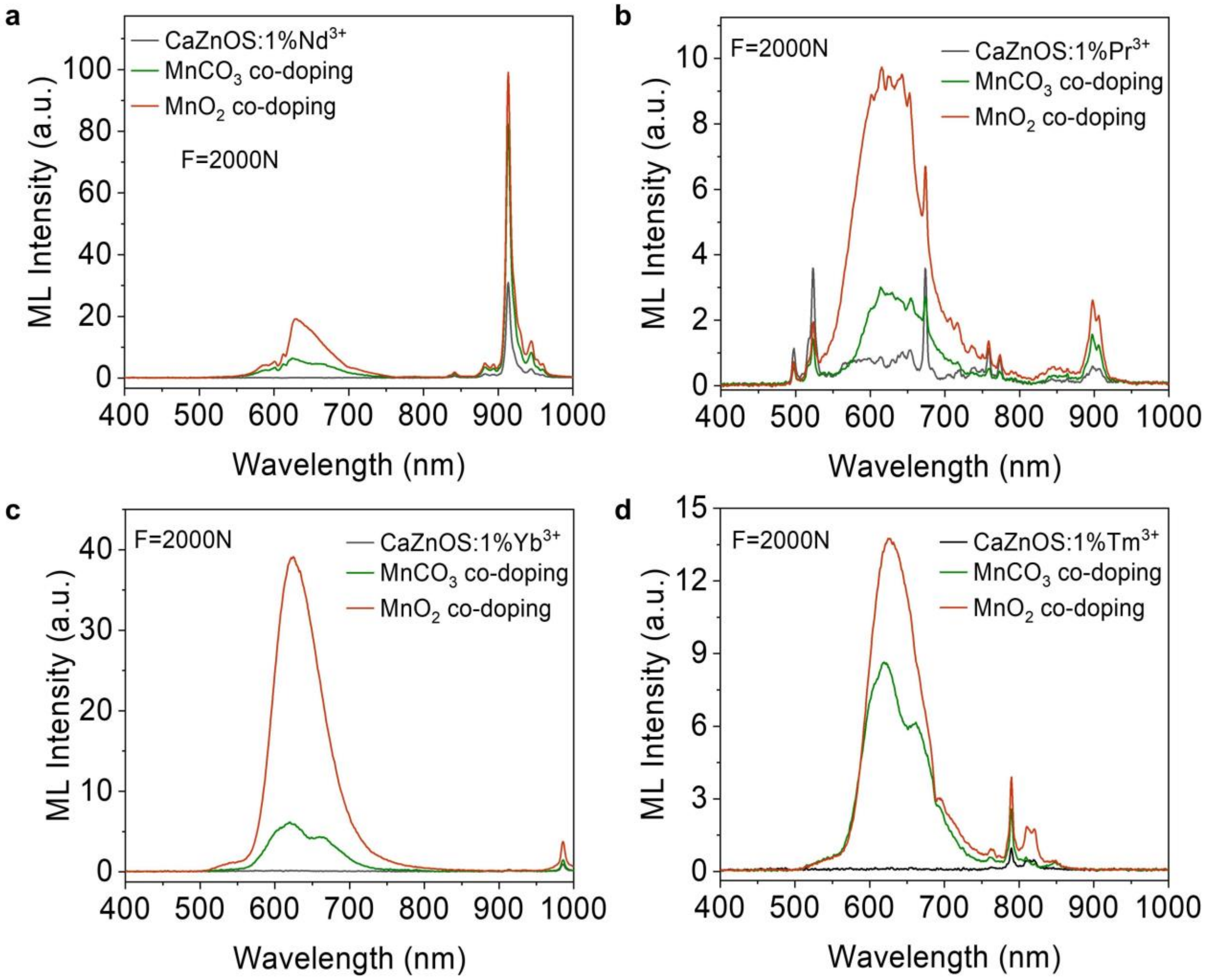

Figure S31. ML spectra of RE irons (Nd^{3+}, Pr^{3+}, Yb^{3+}, Tm^{3+}) co-doped with Mn^{4+} in CaZnOS. And the single-doped RE ions system and the $MnCO_3$/RE co-doping system were set as control groups.

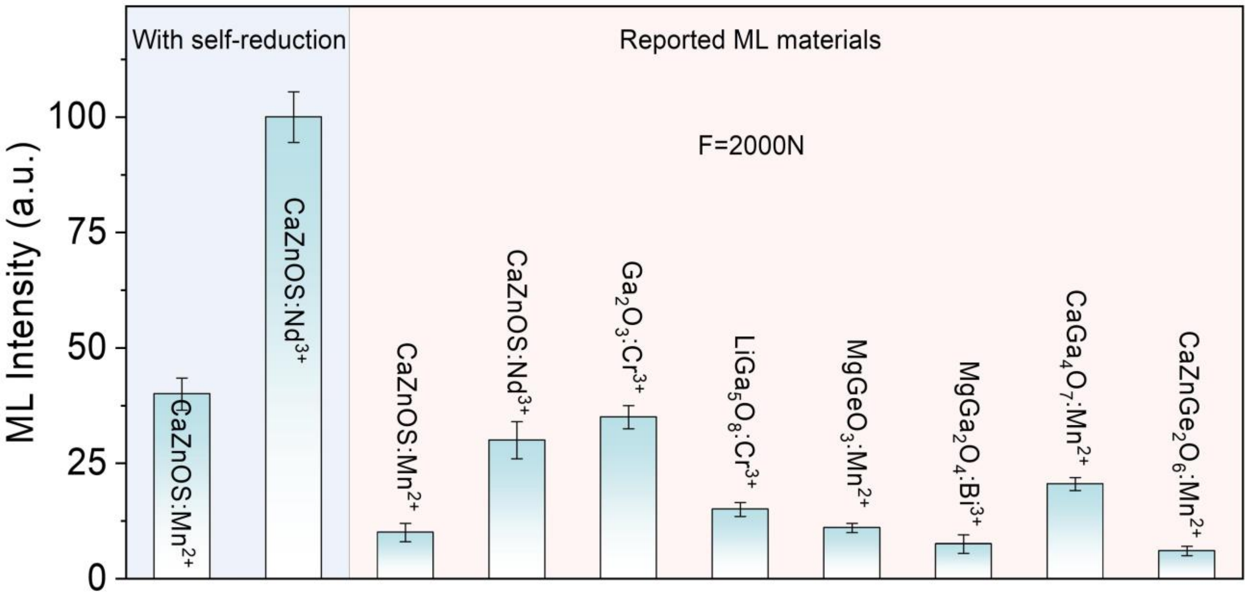

Figure S32. Integral ML intensities of self-reduced CaZnOS-based phosphors compared with those of other reported high-performance ML materials, all measured under a mechanical load of 2000 N.

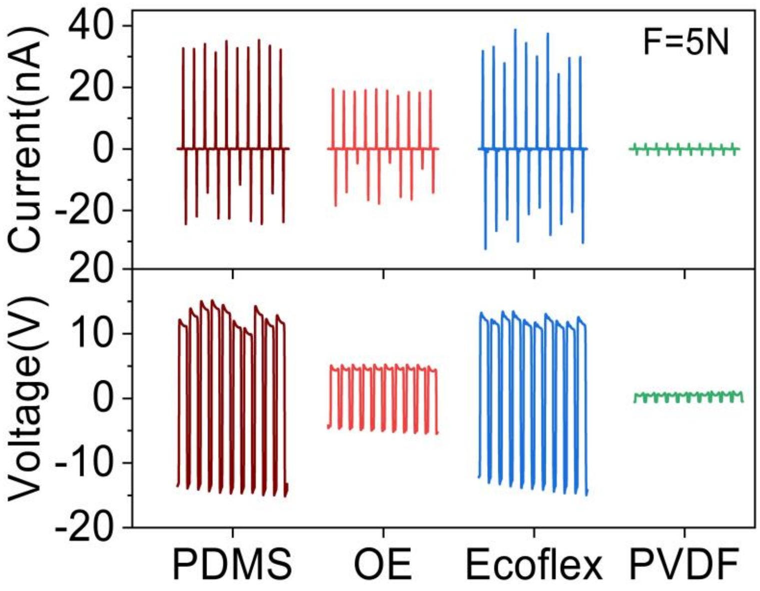

Figure S33. The electrical signals measured when CaZnOS: 1.0%Nd^{3+} with self-reduction phosphor is encapsulated on different substrates under the same pressure.

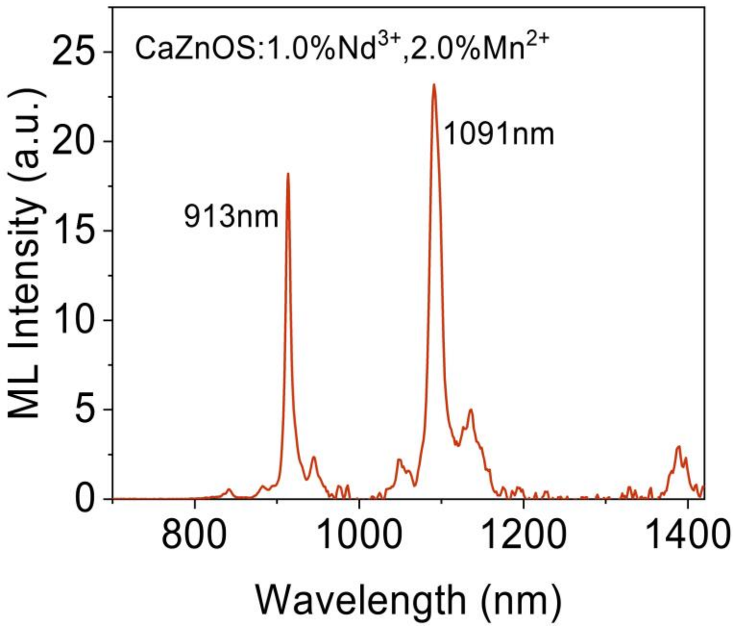

Figure S34. ML spectra (700-1420 nm) of CaZnOS: 1.0%Nd^{3+}, 2.0%Mn^{2+} under a biting force of 5 N.

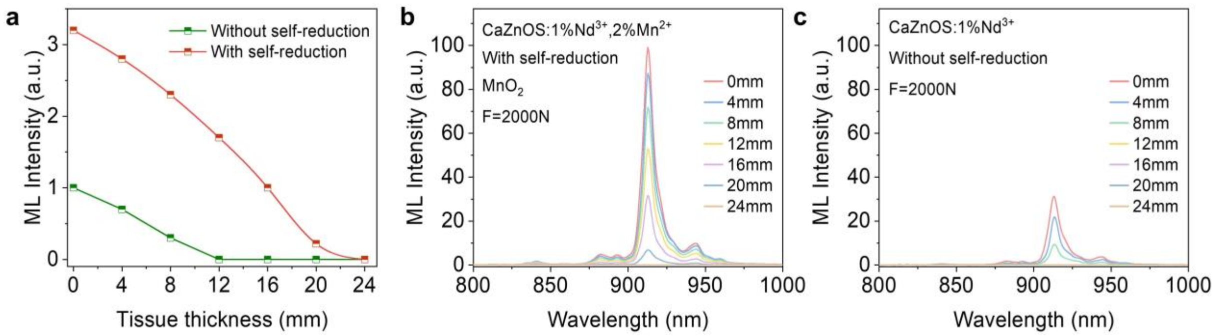

Figure S35. (a) The pork penetration thickness of CaZnOS: Nd^{3+} with and without self-reduction. (b-c) ML spectra measured under the obstruction of pork tissues of different thicknesses with and without self-reduction.

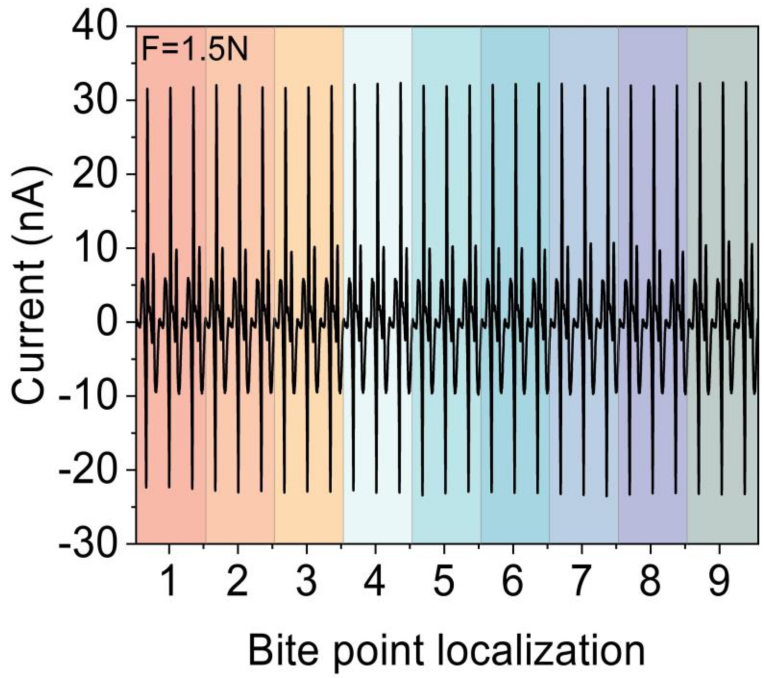

Figure S36. The electrical signals measured when applying the same magnitude of biting force at 9 different occlusal points.

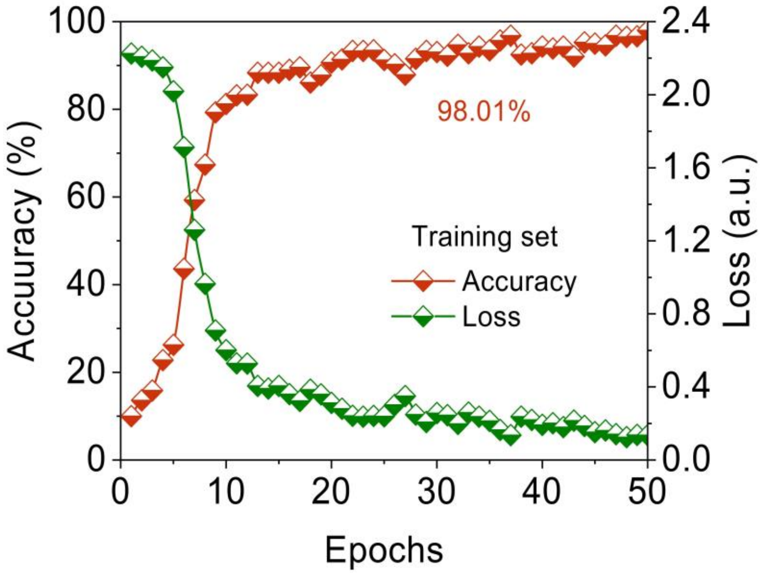

Figure S37. ANN recognition accuracy for occlusal positions vs. training epoch count in the training set.

Table S1 Chemical formula, space group and crystal structure diagram of the previously reported matrices with self-reduction capabilities

**Host**	**Dopant**	**Space group**	**Unit cell parameters**	**Crystal structure**	**Ref**
$SrAl_2O_4$	Eu^{3+}→Eu^{2+}	$P2_12_12_1$ (No. 19)	a= 8.44365 Å<br>b= 8.82245 Å<br>c= 5.15964 Å<br>β=93.411°	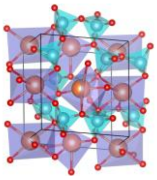	[1]
$MgGeO_3$	Mn^{4+}→Mn^{2+}	Pbca (No. 61)	a= 19.0110 Å<br>b= 9.084 Å<br>c= 5.415 Å	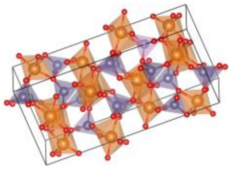	[2]
$CaZnGe_2O_6$	Mn^{4+}→Mn^{2+}	C2/c (No. 15)	10.1659 Å<br>b= 9.0096 Å<br>c= 5.4369 Å<br>β= 105.181°	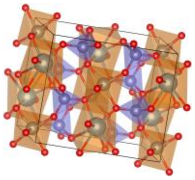	[3]
$MgGa_2O_4$	Bi^{3+}→Bi^{2+}	Fd-3m (No. 227)	a= 8.2781 Å<br>b= 8.2781 Å<br>c= 8.2781 Å	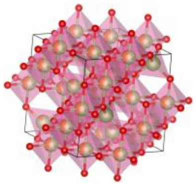	[4]
Na_2ZnSiO_4	Mn^{4+}→Mn^{2+}	$Pna2_1$ (No. 33)	a= 10.759 Å<br>b= 5.313 Å<br>c= 7.211 Å	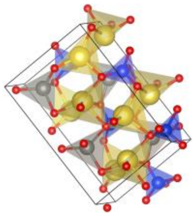	[5]
$CaGa_4O_7$	Mn^{4+}→Mn^{2+}	C2/c (No. 15)	a= 13.065 Å<br>b= 9.079 Å<br>c= 5.6 Å<br>β=105.15°	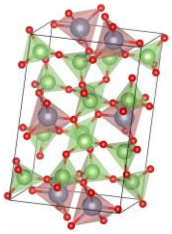	[6]

Table S2 Detailed structure refinement results of CaZnOS: xMn^{2+} (x=0.0%-9.0%) with self-reduction

x value	0.0%	0.5%	1.0%	2.0%	3.0%	5.0%	7.0%	9.0%
Rp (%)	10.0	11.0	11.2	8.91	8.90	11.6	11.1	12.2
Rwp (%)	14.5	14.8	15.2	12.4	12.9	15.4	15.1	16.4
Rexp(%)	10.58	11.57	11.44	10.42	10.63	11.59	11.50	11.47
χ^2(%)	1.88	1.65	1.77	1.42	1.48	1.78	1.72	2.04
a(Å)	3.7556	3.7554	3.7551	3.7546	3.7543	3.7538	3.7536	3.7534
b(Å)	3.7556	3.7554	3.7551	3.7546	3.7543	3.7538	3.7536	3.7534
c(Å)	11.3971	11.3973	11.3974	11.3976	11.3985	11.4	11.4	11.4
V($Å^3$)	139.2166	139.2036	139.1836	139.1563	139.1378	139.1179	139.0893	139.0849
Zn-O(Å)	1.86981	1.88819	1.86074	1.87000	1.86833	1.87610	1.87063	1.89958
Zn-S(Å)	2.36925	2.37292	2.38209	2.36931	2.37110	2.37581	2.36765	2.37027
Ca-O(Å)	2.27590	2.28702	2.28110	2.28865	2.29359	2.28285	2.28671	2.27648
Ca-S(Å)	3.07646	3.03145	3.04757	3.04355	3.03196	3.04113	3.04983	3.04742

The derived lattice parameters a/b (a=b), c, lattice volume V, and bond lengths of CaZnOS: xMn^{2+} (x=0.0%-9.0%) synthesized using MnO_2 are reported. Herein, Rp, Rwp, Rexp, and χ^2 (%) represent the weighted profile reliability factor, pattern reliability factor, Bragg reliability factor, and goodness of fitting, respectively.

Table S3 ICP-OES result of CaZnOS: 3.0%Mn^{2+} with and without self-reduction for all element. The mass fractions of Ca, Zn, O, S, and Mn are as shown in the table.

	Zn (%)	Ca (%)	O (%)	S (%)	Mn (%)
**$MnCO_3$**	25.25	39.34	21.06	13.43	0.92
**MnO_2**	24.95	38.96	20.87	14.24	0.99

Table S4 The Gaussian peak area of the PL spectra of CaZnOS: xMn^{2+} (x=0.5%-9.0%) under 281 nm excitation

CaZnOS: xMn^{2+}	Peak1	Peak2	Peak3
**x=0.5%**	39296002.24	265248015.1	186656010.6
**x=1.0%**	6056878.5	201887764.8	139874530.8
**x=2.0%**	0	121322827.5	83144152.73
**x=3.0%**	0	91290526.89	67923434.91
**x=5.0%**	0	50560414.97	38935881.08
**x=7.0%**	0	24623577.74	20304108.25
**x=9.0%**	0	12429304.85	13510920.9

Table S5 Defect formation energy (E_f) when Mn replaces different lattice sites (Zn1, Zn2, Ca1, Ca2) in CaZnOS matrix.

Model	CaZnOS: Mn_{Zn1}	CaZnOS: Mn_{Zn2}	CaZnOS: Mn_{Ca1}	CaZnOS: Mn_{Ca2}
E_f (eV)	-4.7638	-4.7638	0.1065	0.1065

Equations:

Equation S1:

$$D_r = 100\% \times \frac{[R_m(CN) - R_d(CN)]}{(R_m(CN))} \quad (1)$$

where D_r represents the percentage difference in ionic radii, CN denotes the coordination number, R_m (CN) is the radius of the host cation, and R_d(CN) is the radius of the dopant ion.

Equation S2-4:

$$E_1 = 10B + 5C + 20\alpha \quad (2)$$

$$E_2 = 13B + 5C + 8\alpha \quad (3)$$

$$E_3 = -10D_q + 18B + 6C - \left(\frac{26B^2}{10D_q}\right) + 22\alpha \quad (4)$$

Where E_1 represents the energy level corresponding to the $^4A1$、$^4E(^4G)$，E_2 represents the energy level corresponding to the $^4A_2$ (^4D)，E_1 represents the energy level corresponding to the $^4T_2$ (^4G). The transition energies were obtained from the excitation spectrum. Dq is the crystal field splitting parameter, B is the Racah parameter, and α is the Trees correction term. Substituting the measured data from the experiment, the values of 10Dq/B were calculated to be 11.4 and 14.7 respectively.

Equation S5:

$$Rc \approx 2\left(\frac{3V}{4\pi x_c N}\right)^{\frac{1}{3}} \quad (5)$$

where V represents the unit cell volume, x_c is the critical concentration, and N denotes the number of available cationic sites per unit cell. The calculated R_c value of 51.0 Å and 40.5 Å respectively.

Equation S6:

$$\frac{I}{x} = K\left[1 + \beta(x)^{\frac{\theta}{3}}\right]^{-1} \quad (6)$$

where I is the emission intensity, x represents the activator concentration, and K and β are constants specific to the host lattice under identical excitation conditions. The exponent θ takes values of 6, 8, or 10, corresponding to dipole–dipole, dipole–quadrupole, and quadrupole–quadrupole interactions, respectively.

Equation S7-8:

$$[F(R)h\nu)]^2 = K(h\nu - E_g) \quad (7)$$

$$F(R) = K/S = \frac{(1-R)^2}{(2R)} \quad (8)$$

where R is the reflectance, n = 2 for the direct-bandgap semiconductor CaZnOS, hv is the photon energy, K is a constant, and Eg is the bandgap energy. K is the absorption coefficient, and S is the scattering coefficient.

References

[1] A. M. Achari, V. Perumalsamy, G. Swati, A. Khare, $SrAl_2O_4:Eu^{2+},Dy^{3+}$ Long Afterglow Phosphor and Its Flexible Film for Optomechanical Sensing Application, *ACS Omega* **2023**, 8, 45483.

[2] Y. Xiao, P. Xiong, S. Zhang, Y. Sun, N. Yan, Z. Wang, Q. Chen, P. Shao, M. G. Brik, S. Ye, D. Chen, Z. Yang, Cation-defect-induced self-reduction towards efficient mechanoluminescence in Mn(2+)-activated perovskites, *Mater Horiz* **2023**, 10, 3476.

[3] Z. Wang, Y. Xiao, B. Liu, K. Chen, P. Shao, Z. Chen, P. Xiong, J. Gan, D. Chen, Stress - Induced Multi - Stimulus - Responsive Mechanoluminescence in Mn^{2+} Doped Double Perovskite Compound, *Adv. Opt. Mater.* **2023**, 12, 2301796.

[4] Z. Chen, P. Shao, P. Xiong, Y. Xiao, B. Liu, Z. Wang, S. Wu, D. Jiang, K. Chen, J. Gan, D. Chen, Z. Yang, Visible-to-Near-Infrared Mechanoluminescence in Bi-Activated Spinel Compounds for Multiple Information Anticounterfeiting, *ACS Appl. Mater. Interfaces* **2024**, 16, 35279.

[5] Y. Wei, X. Han, E. Song, Q. Zhang, Photoluminescence and phosphorescence of Mn2+ ion activated green phosphor Na2ZnSiO4:Mn2+ synthesized by self-reduction, *Materials Research Bulletin* **2019**, 113, 90.

[6] P. Zhang, X. Zhao, Z. Jia, J. Dong, T. Liang, Y. Liu, Q. Cheng, L. Ding, L. Wu, D. Peng, Y. Kong, Y. Zhang, J. Xu, High Defect Tolerance Breaking the Design Limitation of Full-Spectrum Multimodal Luminescence Materials, *Adv. Mater.* **2025**, 37, 2411532.
